# Comprehensive Non-Specific Immune and Gene Expression Profiling of FACS-Sorted Hemocyte Subpopulations in *Litopenaeus vannamei* After *Vibrio parahaemolyticus* (AHPND Strain) Infection

**DOI:** 10.3390/ani16203209

**Published:** 2026-10-12

**Authors:** Novi Rosmala Dewi, Ari Widodo, Muhammad Ar Rozzaaq Nugraha, Guan-Lin Lai, Ta-Jeng Yang, Bryan Nathaniel Santoso, Yu-Ru Lin, Yeh-Fang Hu, Fan-Hua Nan

**Affiliations:** 1 Department of Aquaculture, National Taiwan Ocean University, Keelung City 20224, Taiwan; novi.rosmala.d@gmail.com (N.R.D.); nugrahaegha@gmail.com (M.A.R.N.); 21333004@email.ntou.edu.tw (G.-L.L.); 11133001@email.ntou.edu.tw (T.-J.Y.); bryannathaniel22@gmail.com (B.N.S.); linyuru09@gmail.com (Y.-R.L.); 2 School of Materials Science and Engineering, Nanyang Technological University, 50 Nanyang Avenue, Singapore 639798, Singapore; ariwidodo216@gmail.com

**Keywords:** *Litopenaeus vannamei*, hemocyte, prohemocyte, innate immunity, *Vibrio parahaemolyticus*

## Abstract

*Vibrio parahaemolyticus* is the causative agent of acute hepatopancreatic necrosis disease (AHPND) in white shrimp aquaculture, which often causes high mortality. Circulating hemocytes play a central role during vibriosis infection, however, the hemocyte subpopulation specific responses to VP-AHPND infection remain poorly understood. This study used flow cytometry and fluorescence-activated cell sorting (FACS) to characterize four hemocyte subpopulations (H1–H4), including prohemocyte (H1), hyalinocyte (H2), semi-granulocyte (H3), and granulocyte (H4) based on non-specific immune functions and gene-expression profiles after VP-AHPND infection. Differentially hemocyte counts changed dynamically over 48 h, revealing hemocyte subpopulation specific responses to infection, which are H1 peaked during 6 h post infection, H2 increased for most of the period, whereas H3 and H4 declined at 24 and 48 h, respectively. Gene set variation analysis revealed marked early activation (6 hpi) of coagulation and *notch-1* in the H1 subpopulation. The H2 subpopulation showed enrichment of pattern-recognition and coagulation genes, which mostly enhanced in the 12 hpi. Meanwhile, genes associated with prophenoloxidase and antimicrobial peptides (AMPs) were strongly upregulated especially in the late sampling time point (24 and 48 h), coinciding with a decrease in the percentages of their corresponding hemocytes (H3 and H4 subpopulation). These findings reveal that VP infection triggers a coordinated, subpopulation-specific innate immune response instead of a uniform reaction across all hemocytes. By integrating the relative proportions of hemocyte subpopulations, non-specific immune, and gene-expression, this study offers new mechanistic insights into shrimp cellular immunity and highlights key hemocyte subpopulations and pathways as potential targets for enhancing disease resistance in white shrimp aquaculture.

## Simple Summary

This study investigated how different shrimp hemocyte subpopulations respond to *Vibrio parahaemolyticus* (VP-AHPND) infection. Using flow cytometry and FACS, four hemocyte types (H1–H4) were characterized by temporal association with immune function and immune-related gene expression. Results showed distinct and time-dependent responses. Gene analyses revealed specific immune pathway activation, such as coagulation, *notch-1*, antioxidant defense, prophenoloxidase, and antimicrobial peptides varying by subpopulation and infection time point. Overall, VP-AHPND triggers a coordinated, subpopulation-specific immune response, offering insights for improving disease resistance in white shrimp aquaculture.

## 1. Introduction

Invertebrates, including the Pacific white shrimp (*Litopenaeus vannamei*), rely exclusively on innate immune mechanisms in which circulating hemocytes act as the primary effectors of both cellular and humoral defenses [1,2]. Hemocytes are multifunctional immune cells in the hemolymph that coordinate key responses such as phagocytosis, encapsulation, coagulation, and the release of immune effectors [3]. Crustacean hemocytes have been traditionally classified into three hemocyte subpopulations based on morphological and cytochemical characteristics: hyalinocyte (HC), semi-granulocyte (SGC), and granulocyte (GC) [4]. In our previous study, fluorescence-activated cell sorting (FACS) was used to establish an FSC-A/SSC-A gating framework that separated four operationally defined hemocyte populations: H1, prohemocyte (PH); H2, hyalinocyte (HC); H3, semi-granulocyte (SGC); and H4, granulocyte (GC), which are considered a putatively progenitor-like cell type within the shrimp hemolymph [5]. Granulocytes are known to store antimicrobial peptides and contribute strongly to activation of the prophenoloxidase (proPO) system, whereas hyalinocytes are generally regarded as the main phagocytic cell type [5,6]. These hemocyte subpopulations display functional specialization, with granulocytes primarily involved in encapsulation, melanization through the proPO system, and antimicrobial peptide synthesis, while hyalinocytes contribute primarily to phagocytosis and clot formation [7].

Increasing evidence indicates that distinct hemocyte subpopulations respond differently to pathogen infections. For example, in *Fenneropenaeus chinensis*, GC density decreased more rapidly than HC following White Spot Syndrome Virus (WSSV) infection, suggesting that GCs were susceptible to viral infection [1]. In *Macrobrachium rosenbergii*, P53 signaling, DNA repair, and the ROS pathway, were primarily enhanced in HC following infection with decapod iridescent virus 1 (DIVI), suggesting that HC played an important role in apoptosis-driven anti-viral defense [8]. In the context of bacterial infection, previous study demonstrated that HC primarily respond during the early stage of injection by enhancing esterase activity, whereas SGC and GC served as the main functional immune cells executing potent antimicrobial responses through degranulation and elevated ROS/NO production in *Penaeus monodon* following lipopolysaccharide (LPS) stimulation [9].

*Vibrio parahaemolyticus*, the etiological agent of Acute Hepatopancreatic Necrosis Disease (AHPND), poses a catastrophic threat to global shrimp aquaculture, causing mortality rates exceeding 80% harboring plasmid encoding *pirA* and *pirB* toxins, which are central to AHPND pathogenesis [10,11]. Previous study has shown that VP-AHPND infection can rapidly alter hemocyte abundance and immune status [12]. Recent single-cell RNA sequencing studies have also substantially advanced understanding of shrimp hemocyte heterogeneity, revealing transcriptionally distinct hemocyte clusters, potential differentiation trajectories, and heterogeneous responses to infection in *L. vannamei* and *M. rosenbergii* [13,14,15]. Based on single-cell RNA sequencing have shown that prohemocytes, monocytic hemocytes, and granulocytes, including an MH2 macrophage-like phagocytic subpopulation expressing *Nlrp3* and *Casp1* in *L. vannamei* [14], however, this study examined hemocyte classification and phagocytic function rather than infection-induced differentiation. Previous study identified 16 transcriptionally distinct hemocyte clusters during WSSV infection and showed that resting hemocytes became activated and then differentiated into functionally distinct immune subsets, with cluster-specific antiviral responses in *L. vannamei* [1]. Meanwhile, in *M. rosenbergii*, Xiao et al. [13] identified 12 hemocyte clusters during DIV1 infection and found that viral challenge altered hemocyte composition and accelerated differentiation of prohemocytes and semi-granulocytes toward terminally differentiated granulocyte and hyalinocyte populations, particularly an immune-active hyalinocyte subset. Notably, a single-cell transcriptomic atlas of the *L. vannamei* hepatopancreas during AHPND-causing *V. parahaemolyticus* infection was recently generated, identifying epithelial, myocyte, and hemocyte populations and cluster-specific immune and metabolic responses [16]. However, this tissue-based study did not characterize the temporal, subpopulation-resolved responses of circulating hemocytes during the acute phase of VP-AHPND infection.

Our previous study established an FSC-A/SSC-A-based FACS gating framework for four operationally defined hemocyte populations in *L. vannamei*: H1, prohemocyte-like cells (PH); H2, hyalinocyte-like cells (HC); H3, semi-granulocyte-like cells (SGC); and H4, granulocyte-like cells (GC) [5]. The present study extends that descriptive classification by examining the temporal immune responses of these FACS-defined populations during challenge with an AHPND-associated *V. parahaemolyticus* strain. Specifically, we combined relative proportions of H1–H4 hemocyte subpopulations with qPCR-based gene expression analysis of selected genes associated with pathogen recognition and immune-effector functions. The selected genes were included to assess hemocyte responses related to pathogen recognition and downstream immune-effector activity (AMPs and proPO-related genes). Sampling at 6, 12, 24, and 48 hpi was designed to capture the hemocyte responses during the acute phase of VP-AHPND challenge. Although previous studies have shown that *pirA* and *pirB* toxins can induce apoptosis, the present study focuses on the functional and transcriptional responses of hemocyte subpopulations rather than directly evaluating apoptosis. We hypothesize that hemocyte subpopulations differ in both timing and type of immune responses following *V. parahaemolyticus* strain AHPND infection. Subsequent experiments were conducted to examine changes in hemocyte subpopulation percentages and to evaluate functional immune responses at different time points, thereby providing a framework for developing hemocyte-targeted strategies to mitigate AHPND in shrimp aquaculture.

## 2. Materials and Methods

### 2.1. Experimental Animals

*L. vannamei* (20.35 ± 3.63 g) were obtained from Aquatic Animal Center at National Taiwan Ocean University, Taiwan. Shrimps were reared in seawater tank with aeration and filtration systems for one week of acclimation. The shrimps were cultured under optimal conditions at 25 °C and 33‰ salinity. Feeding was performed three times daily (08:00, 15:00, and 21:00) during acclimation at a rate of 5% body weight per day. Prior to the experiment 5 individual shrimps were randomly selected from the acclimated population for health screening [17,18,19]. Hemocyte and hepatopancreas samples were collected from each shrimp, and total DNA was extracted using TRIzol reagent (Invitrogen, Carlsbad, CA, USA). The samples were screened for *Enterocytozoon hepatopenaei* (EHP) and the AHPND-associated *Vibrio* virulence genes p*irA* and *PirB* by PCR (Supplementary Figure S1). All screened shrimp tested negative for EHP and for *pirA*/*pirB* amplification.

### 2.2. Vibrio Parahaemolyticus Challenge (AHPND Strain, VP-AHPND)

#### 2.2.1. Bacterial Preparation

The preparation of *V. parahaemolyticus* (VP) AHPND strain was followed by the previously described [20]. The VP strain AHPND isolate from diseased shrimp, stored at −80 °C, was thawed immediately before use. Prior to the challenge experiment, the isolate was streaked onto CHROMagar™ Vibrio agar to purify the culture. The presence of AHPND-associated *pirA* and *pirB* genes was confirmed by PCR using the Vp*pirA*-284 and Vp*PirB*-392 primer sets reported by Restrepo et al. [21]. The PCR products were separated by agarose-gel electrophoresis, yielding the expected 284-bp *pirA* and 392-bp *pirB* amplicons. After preparation, a single colony was inoculated into 200 mL of tryptic soy broth (TSB; Neogen Corporation, Lansing, MI, USA) supplemented with 2% NaCl and incubated at 28 °C with shaking at 150 rpm for 16 h. The resulting culture was harvested by centrifugation at 4 °C and 5000× *g* for 5 min using an Allegra X-30 benchtop centrifuge (Beckman Coulter, Brea, CA, USA), and the supernatant was discarded. The bacterial pellet was washed three times with 1× phosphate-buffered saline (PBS; GeneStar, Kaohsiung, Taiwan) and finally resuspended in 1× PBS. To determine the bacterial challenge dose, an OD_600_ CFU calibration curve was established for the isolate using serial dilution and viable colony enumeration on agar. The calibration equation was CFU/mL = 1 × 10^8^ × OD_600_ − 746,277 (R^2^ = 0.9983). The bacterial culture was initially adjusted to an OD_600_ of 1.0, corresponding to approximately 1.2 × 10^8^ CFU/mL according to the OD_600_ calibration curve. The suspension was subsequently diluted approximately 10-fold with 1× PBS sterile to obtain a final challenge inoculum of 1 × 10^7^ CFU/mL, which was used for shrimp infection. The optical density of the suspension was measured using a UV-1280 UV-Vis spectrophotometer (Shimadzu, Kyoto, Japan).

#### 2.2.2. Infection with *V. parahaemolyticus*

A total of 180 healthy shrimp (20.35 ± 3.63 g) were randomly allocated to a 1× PBS control group (PBS group) and a VP-AHPND challenge group (VP group), with 90 shrimp per group. Each group consisted of three replicate tanks, with 30 shrimp stocked per tank. Shrimp in the PBS group received an intraperitoneal injection of 20 µL sterile 1× PBS. Shrimp in the VP group received an intraperitoneal injection of 20 µL VP-AHPND suspension at 1.0 × 10^7^ CFU/mL, a challenge dose previously reported to approximate the LD_50_ [5]. Shrimp were sampled at 0, 6, 12, 24, and 48 h post-injection (hpi). The 0 h time point was defined as immediately before intraperitoneal injection of PBS or the VP-AHPND suspension and represented the pre-challenge baseline. At each time point, three shrimp were collected from each of three replicate tanks per group, yielding nine shrimp per group per time point and 45 shrimp per group across all sampling time points. The remaining shrimp were maintained to ensure sufficient animals were available for the scheduled sampling design. Shrimp were monitored during the experiment, and any moribund or dead shrimp were removed according to institutional animal-care and biosafety procedures. Some mortality was observed following VP-AHPND challenge. However, daily mortality was not systematically recorded because the present study focused on hemocyte-subpopulation dynamics and immune-related responses. Therefore, standardized survival data were unavailable, and a Kaplan-Meier survival analysis could not be performed. This is a limitation of the present study. Following bacterial challenge, shrimp were fasted for the initial 24 h to minimize variation in water quality and metabolic activity during the early response period. Thereafter, feeding was resumed, and shrimp were fed three times daily at 08:00, 15:00, and 21:00 at a rate of 5% body weight per day until the end of the experiment. After challenge, shrimp were maintained under the same conditions (25 °C, 33‰ salinity) for 48 h until the end of the experiment.

### 2.3. Hemocyte Sampling and Flow Cytometry Sorting FACS

Hemocyte samples were collected at 0, 6, 12, 24, and 48 h post-injection (hpi). Hemolymph was withdrawn from the ventral sinus using a 1-mL syringe fitted with a 25-gauge needle and immediately mixed with anticoagulant (AC) buffer (30 mM trisodium citrate, 0.34 M sodium chloride, 10 mM EDTA, 0.12 M glucose at pH 7.4 and 780 mOsm/kg) in a 1:9 ratio. Prior to cell sorting, hemocytes were adjusted to a concentration of 2 × 10^6^ cells/mL and resuspended in AC buffer supplemented with 2% fetal bovine serum (FBS). Hemocyte subpopulations were collected using BD FACSAria II flow cytometer (Becton Dickinson, San Jose, CA USA). Cytograms were generated using forward scatter area (FSC-A) and side scatter area (SSC-A) following previously described [5], with the flow rate adjusted to keep the total event rate below 4 (≤0.5 psi above sheath pressure). A total hemocyte concentration of 5 × 10^5^ cells/mL was prepared for each subpopulation, as described in the previous study [22].

### 2.4. Parameter Analysis

#### 2.4.1. Gating Strategy and Relative Proportions of Hemocyte Subpopulations

**Figure 1:**
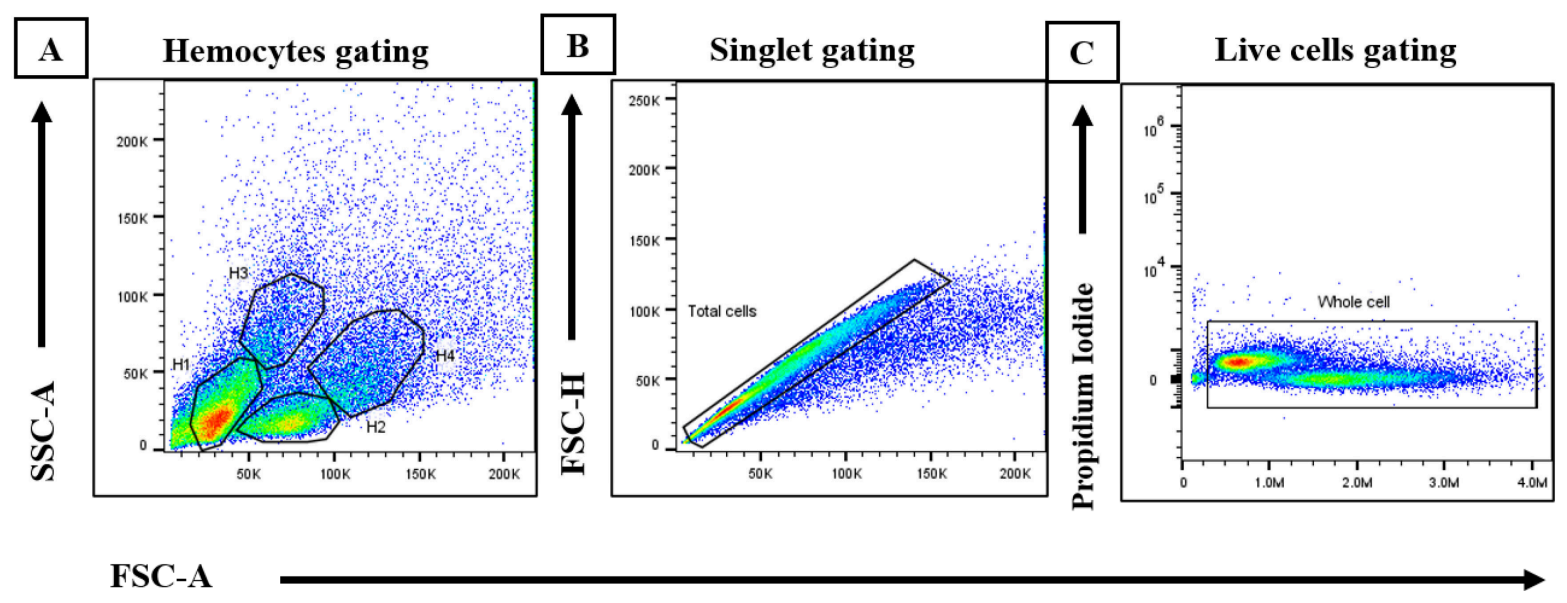
Gating strategy for flow cytometric analysis. Example for hemocyte subpopulations. (**A**) Gate of valid H1, H2, H3 and H4 (FSC-A and SSC-A); (**B**) Gate of singlets in FSC-A/FSC-H; (**C**) Propidium iodide (PI)-negative hemocytes selected from the FSC-A/SSC-A-gated population shown in panel (**A**).

Firstly, the H1–H4 hemocyte subpopulations were identified using the previously characterized FSC-A/SSC-A gating criteria described by Dewi et al. (2025) [5], which were applied consistently to the PBS and VP samples at all sampling time points. The relative proportions of hemocyte subpopulations were determined at 0, 6, 12, 24, and 48 h post-injection (hpi) using the method previously described [5]. Hemolymph was mixed with AC buffer at a 1:4 ratio, and the hemocyte density was adjusted to 2 × 10^6^ cells/mL before analysis on a BD FACSAria II flow cytometer (Becton Dickinson, San Jose, CA, USA). Cytograms were obtained using FSC-A and SSC-A parameters, and 10,000 events were collected for each sample to generate dot plots of cell size (FSC) and granularity (SSC). H1–H4 subpopulations were defined using FSC-A/SSC-A gates, as illustrated in Figure 1. The relative proportion of each subpopulation was calculated as the percentage of events within the total gated hemocyte population. Because hemocyte suspensions were normalized before flow-cytometric analysis, the data represent relative subpopulation distributions and do not represent total hemocyte counts or absolute abundances of H1–H4 cells in the original hemolymph samples.

#### 2.4.2. Non-Specific Immune Response

Phagocytosis activity in hemocyte subpopulations was evaluated following the protocol from Dewi et al. (2025) [5], with minor modifications. After sorting, hemocytes (5 × 10^5^ cells/mL; 1000 µL) were centrifuged at 800× *g* for 20 min at 4 °C, the supernatant was discarded, and the cells were resuspended in MCHBSS. Carboxylate modified yellow green fluorescent latex beads (1 µm diameter; Invitrogen, Carlsbad, CA, USA) were then added (1 µL, 1 × 10^6^ beads), and the suspension was incubated for 30 min at room temperature on a rotary mixer (20 rpm) in the dark. After incubation, cells were washed twice by centrifugation (800× *g*, 20 min, 4 °C) and resuspended in Leibovitz’s L-15 medium (L15; Gibco, Thermo Fisher Scientific, Grand Island, NY, USA) to remove excess beads. Fluorescence was recorded on an Attune CytPix Flow Cytometer (Thermo Fisher Scientific, Waltham, MA, USA) using 505 nm excitation and 515 nm emission.

Phenoloxidase (PO) activity was determined spectrophotometrically by monitoring dopachrome formation from L-DOPA, following a previously described protocol [23], with minor modifications. Hemocyte subpopulations were centrifuged at 800× *g* for 20 min at 4 °C. The supernatant was discarded, and the cells were resuspended in 1 mL cacodylate citrate buffer (0.01 M sodium cacodylate, 0.45 M sodium chloride, 0.01 M trisodium citrate, pH 7.0) and centrifuged again. The pellet was then resuspended in 200 µL cacodylate buffer (0.01 M sodium cacodylate, 0.45 M sodium chloride, 0.01 M calcium chloride, 0.26 M magnesium chloride, pH 7.0) and split into two 100 µL aliquots for total and background PO activity measurements. For total PO activity, 100 µL of the cell suspension was incubated with 50 µL trypsin (1 mg/mL; Sigma-Aldrich, St. Louis, MO, USA) for 10 min at room temperature, whereas background activity was assessed by incubating 100 µL of cells with 50 µL cacodylate buffer for 10 min. Afterward, 50 µL L-DOPA (3 mg/mL; Sigma-Aldrich, St. Louis, MO, USA) was added to each tube, which was then incubated for 15 min at room temperature, followed by dilution with 400 µL cacodylate buffer. Absorbance was read at 492 nm using a Hitachi U-2000 spectrophotometer (Tokyo, Japan), and PO activity was calculated using the following formula:

PO activity (OD. 492 nm) = OD of sample − OD of background

Superoxide anion (O_2_^−^) production in hemocytes was evaluated using the NBT reduction assay, following a previously described method [23], with minor modifications. After sorting, each hemocyte subpopulation in different groups were incubated in a 96-well plate with either 100 µL zymosan (1 mg/mL; Sigma-Aldrich, St. Louis, MO, USA) to induce O_2_^−^ production or 100 µL MCHBSS as an unstimulated control for 30 min at room temperature. After incubation, the supernatant was removed, and cells were treated with 100 µL NBT solution (0.3%; Panreac, Darmstadt, Germany) for 30 min at room temperature. The reaction was stopped by discarding the NBT solution and adding 100 µL methanol; after 5 min, methanol was removed, the wells were washed three times with 100 µL 70% methanol, and then air-dried for 30 min. Formazan crystals were solubilized with 120 µL 2 M KOH (Sigma-Aldrich, St. Louis, MO, USA) and 140 µL DMSO (Riedel-de Haen, Seelze, Germany), and absorbance was read at 630 nm using a Hitachi U-2000 spectrophotometer (Tokyo, Japan). Superoxide production was calculated using the following formula:

O_2_^−^ production (%) = (OD of sample − OD of background)/(OD of background) × 100

#### 2.4.3. Gene Expression

Total RNA was extracted from each hemocyte subpopulation for every group at each time point. Firstly, hemocytes were collected using FACS sorting, the samples were centrifuged at 800× *g* for 20 min at 4 °C. The supernatant was then removed, and the resulting pellet was rinsed three times with 1000 µL of AC buffer. A total of 500 μL of Trizol reagent (Invitrogen, Carlsbad, CA, USA) was added to each sample and kept at −80 °C before extraction. Total RNA was isolated using TRIzol reagent following the manufacturer’s instructions, and any remaining genomic DNA was eliminated with RNase-free DNase I (Bionovas, Toronto, ON, Canada) [23,24]. RNA purity and concentration were assessed using a SpectraMax QuickDrop Micro-Volume Spectrophotometer (Molecular Devices, San Jose, CA, USA). First-strand cDNA was synthesized from 1 µg of total RNA using a commercial reverse transcription kit following the manufacturer’s instructions (Bionovas, Toronto, ON, Canada). The qPCR reaction mixture (20 µL) contained 1 µL of cDNA, 10 µL of SYBR Green Supermix with ROX, 0.4 µL of forward primer (10 µM), 0.4 µL of reverse primer (10 µM), and 8.2 µL of DEPC-treated sterile filtered water. The qPCR reaction was performed using the QuantStudio1 Real-Time PCR machine (Applied Biosystems, Thermo Fisher Scientific, South San Francisco, CA, USA). The thermal cycling conditions began with an initial step at 50 °C for 2 min, followed by an enzyme activation step at 95 °C for 10 min. This procedure was followed by cycling (40 cycles) at 95 °C for 15 s, then at 60 °C for 1 min. The melting curve stage was initiated at 95 °C for 15 s, followed by 60 °C for 1 min, and then repeated with another 95 °C for 15 s.

The expression of functional genes was measured, including elongation factor-1α (*ef1α*), lipopolysaccharide and β-1,3-glucan-binding protein (*lgbp*), c-type lectin 2 (*ctl-2*), prophenoloxidase 1 and 2 (*propo-1* and *propo-2*), serine proteinase (*sp*), superoxide dismutase (*sod*), glutathione peroxidase (*gpx*), clotting protein (*cp*), transglutaminase (*tgase*), peroxinectin (*px*), crustin (*cru*), anti-lipopolysaccharide factor (*alf*), penaeidin 2 and 3 (*pen-2-3*), and neurogenic locus notch homolog protein 1 (*notch-1*) [23]. The primers are listed in Table 1. Relative mRNA expression levels were calculated using the 2^−ΔΔCt^ method, normalizing target genes to the reference gene (*ef1α*) and expressing results as fold-change relative to the control group, with data provided as means ± standard deviations (means ± SD) [25,26,27].

### 2.5. Data Analysis

All statistical analyses and data visualizations were performed using GraphPad Prism version 10. For each treatment at each time point, three shrimps were sampled from each of three independent replicate tanks. To avoid pseudo-replication, measurements from the three shrimps sampled from the same tank were averaged before analysis. Therefore, the tank was treated as the experimental unit (*n* = 3 independent tanks per treatment per time point; N = 9 shrimp sampled per treatment per time point). Data are presented as mean ± standard deviation (SD). Because the primary objective of the study was to compare the PBS and the VP group at each predefined post-injection time point, relative proportions of hemocyte subpopulations (H1–H4), non-specific immune parameters, and gene-expression levels were analyzed using independent two-sided Student’s t-tests based on tank-level mean values. Prior to t-test analysis, normality and homogeneity of variances were assessed using the Shapiro-Wilk test and Levene’s test, respectively, in IBM SPSS Statistics version 26 (IBM Corp., Armonk, NY, USA). Differences were considered statistically significant at *p* < 0.05; significant differences are indicated by an asterisk (*).

## 3. Results

### 3.1. Gating Strategy and Relative Proportions of Hemocyte Subpopulations

The flow-cytometry gating strategy used for hemocyte analysis is shown in Figure 1. Hemocyte subpopulations H1, H2, H3, and H4 were identified based on their FSC-A and SSC-A characteristics. Singlet cells were selected using FSC-A versus FSC-H to exclude cell aggregates and doublets, and viable cells were identified by propidium iodide staining. This gating strategy was applied to all PBS- and VP-challenged samples at each sampling time point.

**Figure 2:**
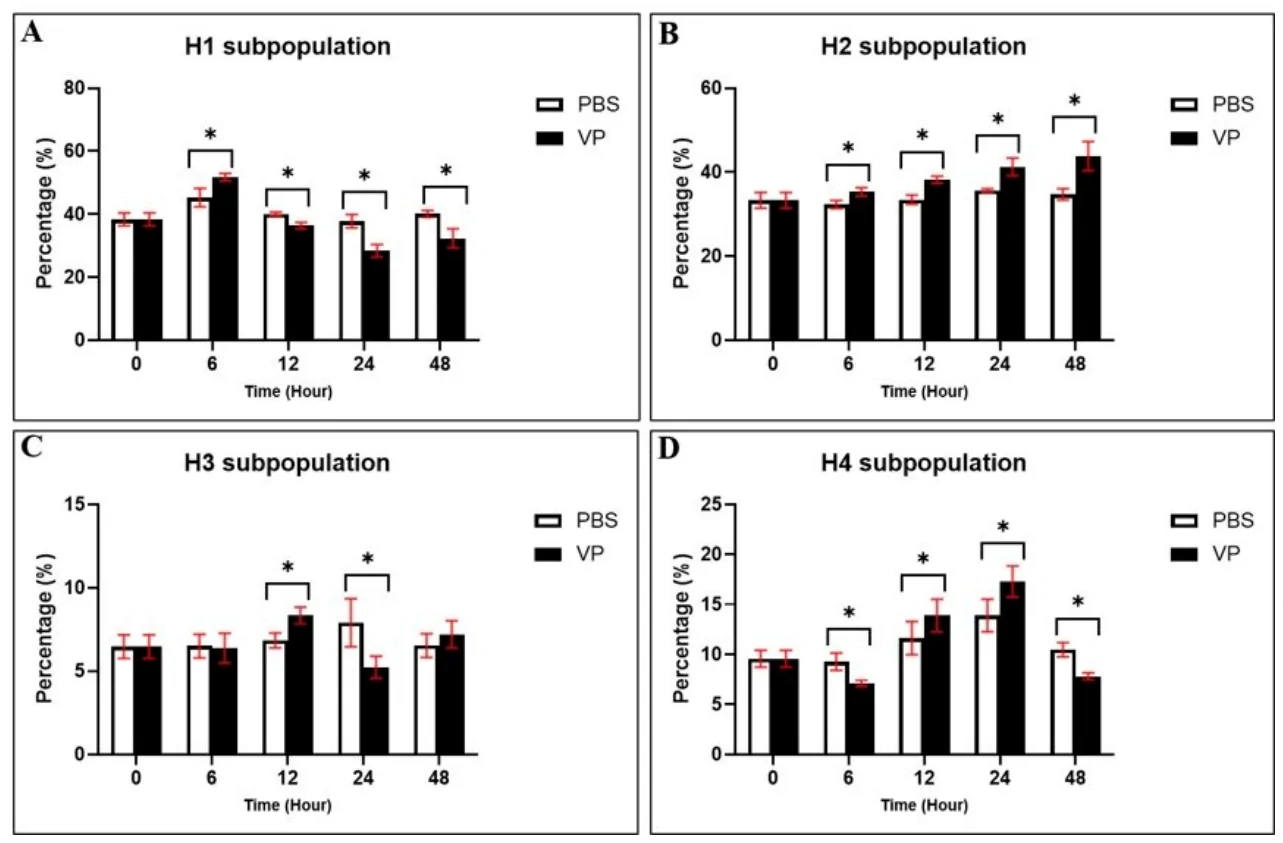
Relative proportions of hemocyte subpopulations in *Litopenaeus vannamei* following *Vibrio parahaemolyticus* and PBS injection over 48 h. (**A**) Hemocyte subpopulation 1 (H1); (**B**) Hemocyte subpopulation 2 (H2); (**C**) Hemocyte subpopulation 3 (H3); (**D**) Hemocyte subpopulation 4 (H4). Data are presented as the mean ± SD of tank-level means (*n* = 3 independent tanks per treatment at each time point). Three shrimp were sampled from each tank (*N* = 9 shrimp per treatment at each time point) independent tanks per treatment at each time point). At 0 hpi, the data represent a baseline collected before PBS or VP injection; the same baseline values are shown for both groups for visual comparison. The red error bars represent the standard deviation (SD). Asterisk indicate significant differences at * *p* < 0.05.

Relative proportions of hemocyte subpopulation of *L. vannamei* changed markedly over the 48 h period following injection with *V. parahaemolyticus* (Figure 2). The proportion of the H1 subpopulation in the *V. parahaemolyticus* (VP) group differed significantly from that in the PBS group at 6–48 h post-injection (*p* < 0.05). Specifically, the H1 proportion was significantly higher than that in PBS group at 6 h (*p* < 0.01), followed by a declining trend at 12, 24, and 48 h, where it became significantly lower than that in the PBS group (*p* < 0.05). (Figure 2A). The H2 subpopulation in the VP group was significantly elevated (*p* < 0.05) compared with PBS group at all time points, displaying a gradual increase over time (Figure 2B). The H3 subpopulation exhibited a delayed response, showing significant differences between the VP and PBS groups at 12 and 24 h post-injection (*p* < 0.05). Specifically, the proportion of H3 increased at 12 h and subsequently decreased at 24 h compared with the PBS group (Figure 2C). The H4 subpopulation in the VP group differed significantly from that in the PBS group at all time points examined (*p* < 0.05). The proportion of H4 decreased at 6 h post-injection, increased at 12–24 h, and then declined again by 48 h (Figure 2D). Overall, the VP group showed significant differences from the PBS group across all hemocyte subpopulations, but with distinct time-dependent patterns, suggesting that VP challenge affected specific subsets at particular stages rather than causing uniform changes over the whole experimental period. The main phenomenon was that H1 increased sharply early in the infection, whereas H4 tended to decrease. By the end of the experimental period, only H2 remained elevated, while both H1 and H4 had declined relative to the PBS group.

### 3.2. Non-Specific Immune Response

**Figure 3:**
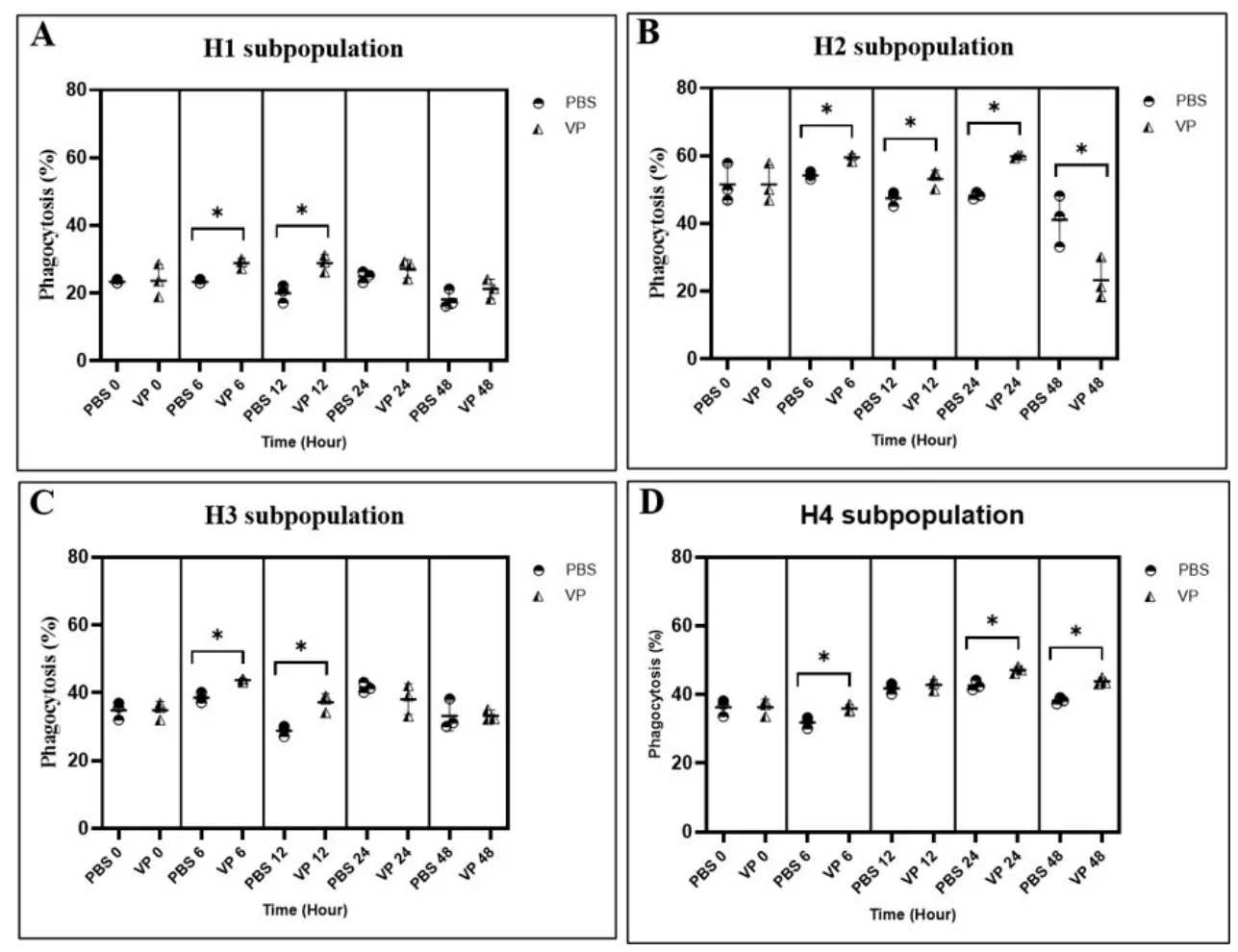
Phagocytosis activity in hemocytes of *L. vannamei* following *V. parahaemolyticus* and PBS injection over 48 h. (**A**) Hemocyte subpopulation 1 (H1); (**B**) Hemocyte subpopulation 2 (H2); (**C**) Hemocyte subpopulation 3 (H3); (**D**) Hemocyte subpopulation 4 (H4). Three shrimp were sampled from each tank, and the mean value of the three shrimp within each tank was used for analysis. Each symbol represents one tank-level mean. Horizontal lines indicate the mean, and error bars indicate the standard deviation (SD) of three independent tanks (*n* = 3 independent tanks per treatment at each time point; *N* = 9 shrimp per treatment at each time point). Asterisks indicate significant differences between the PBS and VP groups at the same time point (* *p* < 0.05).

Phagocytosis activity of hemocyte subpopulations in *L. vannamei* showed distinct responses to *V. parahaemolyticus* challenge over the 48 h period (Figure 3). The H1 and H3 subpopulations showed the same trend; phagocytosis activity in the VP group was significantly increased (*p* < 0.05) from the PBS group at 6–12 h, following no significant differences were observed at 24–48 h (Figure 3A,C). In contrast, the H2 subpopulation displayed consistently elevated phagocytosis activity (*p* < 0.05) in the VP group than in PBS at 6, 12, and 24 h, but significantly declined at 48 h (*p* < 0.001) (Figure 3B). For H4, phagocytic activity in the VP group was significantly higher than in the PBS group at 6, 24, and 48 h (*p* < 0.05) (Figure 3D). Overall, H1 and H3 showed increased activity mainly during the early time of infection, whereas H2 and H4 were more active across multiple time points, with H2 showed the most significant difference in phagocytosis compared with the PBS group across the entire sampling period.

**Figure 4:**
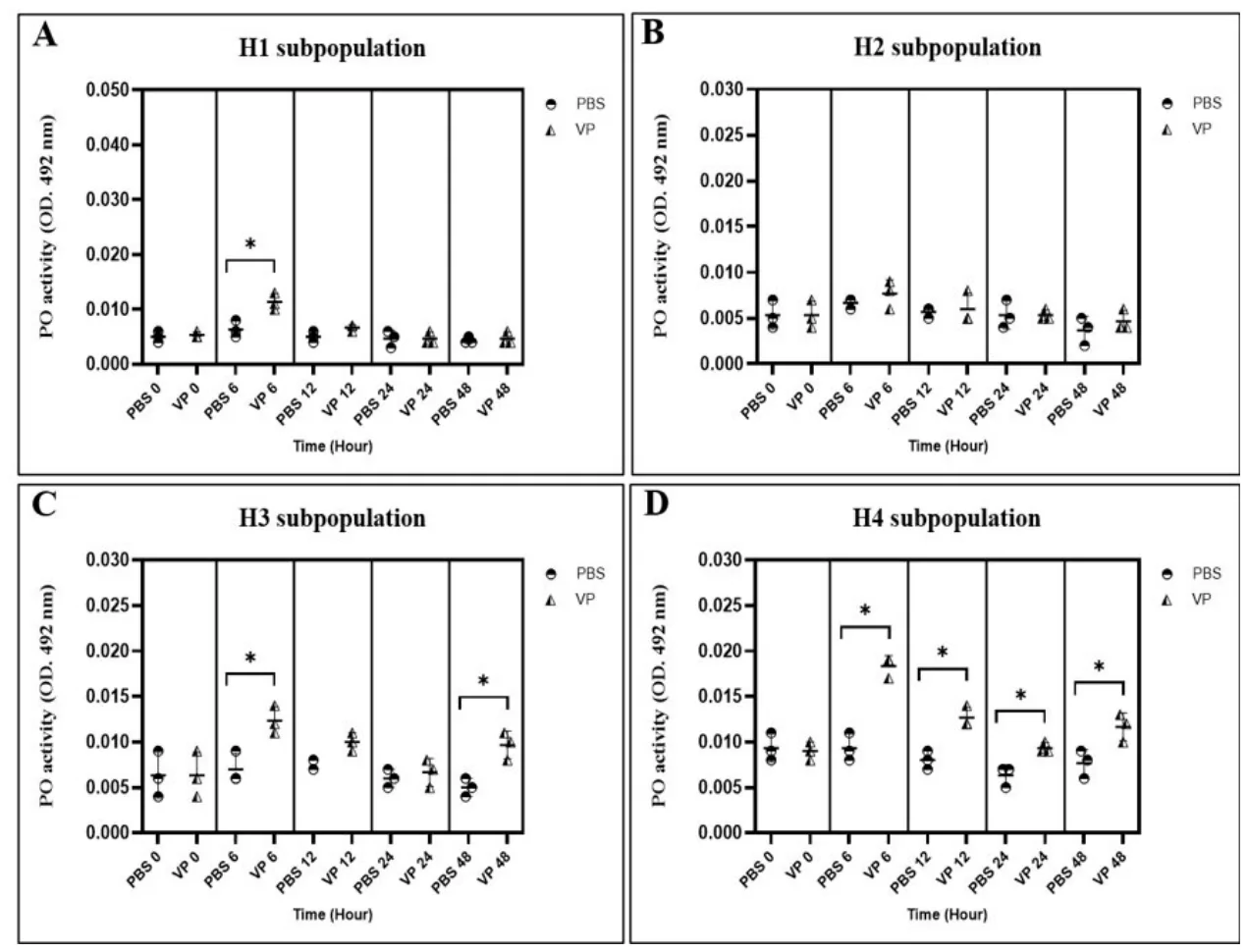
Phenoloxidase activity in hemocytes of *L. vannamei* following *V. parahaemolyticus* and PBS injection over 48 h. (**A**) Hemocyte subpopulation 1 (H1); (**B**) Hemocyte subpopulation 2 (H2); (**C**) Hemocyte subpopulation 3 (H3); (**D**) Hemocyte subpopulation 4 (H4). Three shrimp were sampled from each tank, and the mean value of the three shrimp within each tank was used for analysis. Each symbol represents one tank-level mean. Horizontal lines indicate the mean, and error bars indicate the standard deviation (SD) of three independent tanks (*n* = 3 independent tanks per treatment at each time point; *N* = 9 shrimp per treatment at each time point). Asterisks indicate significant differences between the PBS and VP groups at the same time point (* *p* < 0.05).

Phenoloxidase (PO) activity associated with different hemocyte subpopulations of *L. vannamei* was modulated by *V. parahaemolyticus* challenge in a time- and hemocyte subpopulation-dependent manner (Figure 4). In the H1 subpopulation, PO activity was significantly higher in the VP group than the PBS group at 6 h (*p* < 0.05). No significant differences were observed at later time points (Figure 4A). In H2, PO activity showed only minor fluctuations, with no significant differences between VP and PBS groups across the sampling period (Figure 4B*)*. The H3 subpopulation displayed significantly elevated PO activity in the VP group at 6 and 48 h (*p* < 0.05) (Figure 4C). In H4, VP injection led to significantly higher (*p* < 0.05) PO activity than PBS at 6–48 h (Figure 4D), suggesting that this subpopulation contributes prominently to PO-mediated responses at multiple stages of infection.

**Figure 5:**
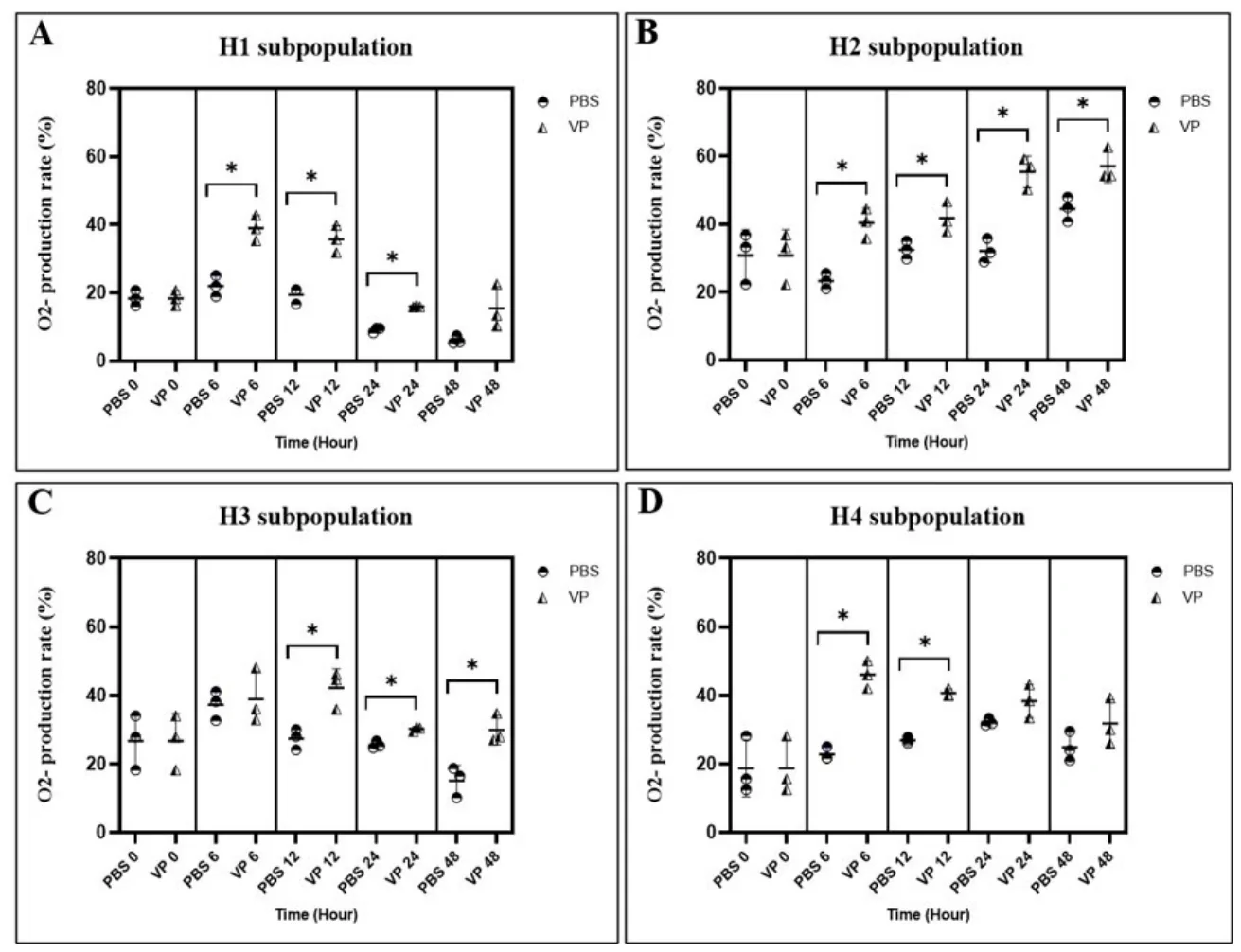
Superoxide anion (O_2_^−^) production of hemocytes of *L. vannamei* following *V. parahaemolyticus* and PBS injection over 48 h. (**A**) Hemocyte subpopulation 1 (H1); (**B**) Hemocyte subpopulation 2 (H2); (**C**) Hemocyte subpopulation 3 (H3); (**D**) Hemocyte subpopulation 4 (H4). Three shrimp were sampled from each tank, and the mean value of the three shrimp within each tank was used for analysis. Each symbol represents one tank-level mean. Horizontal lines indicate the mean, and error bars indicate the standard deviation (SD) of three independent tanks (*n* = 3 independent tanks per treatment at each time point; *N* = 9 shrimp per treatment at each time point). Asterisks indicate significant differences between the PBS and VP groups at the same time point (* *p* < 0.05).

O_2_^−^ production in hemocytes increased markedly after *V. parahaemolyticus* challenge and differed among hemocyte subpopulations (Figure 5). In the H1 subpopulation, the VP group showed significantly higher O_2_^−^ production than the PBS group at 6–24 h (*p* < 0.05) with a highest O_2_^−^ production at 6 h (Figure 5A). In H2, O_2_^−^ production in the VP group was significantly elevated at 6, 12, 24 and 48 h (*p* < 0.05) (Figure 5B), showing a progressive increasement over time and the most sustained enhancement among all hemocyte subpopulations. The H3 subpopulation exhibited significantly higher O_2_^−^ production in the VP group at 12, 24, and 48 h compared to the PBS group (*p* < 0.05) (Figure 5C). For H4, VP injection led to significantly greater O_2_^−^ production than PBS group at 6 and 12 h (*p* < 0.05) (Figure 5D), after which the activity remain the same with the PBS group, suggesting that H4 contributes mainly increase O_2_^−^ production in the early infection.

### 3.3. Gene Expression

**Figure 6:**
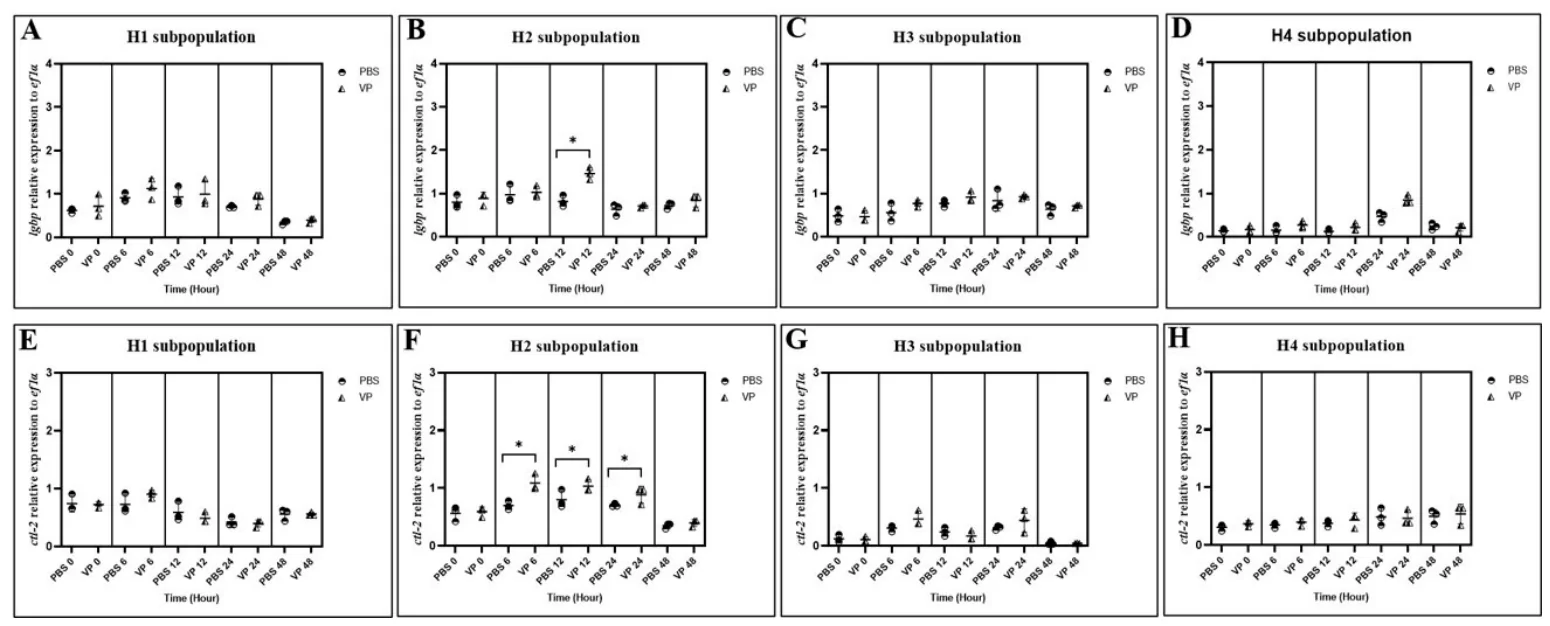
Expression of pattern recognition receptor (PRR) genes in hemocytes of *L. vannamei* following *V. parahaemolyticus* and PBS injection over 48 h. Expression levels of (**A**–**D**) Lipopolysaccharide and β-1,3-glucan-binding protein (*lgbp*) and (**E**–**H**) C-type lectin-2 (*ctl-2*) in H1, H2, H3, and H4, respectively. H1–4: hemocyte subpopulation 1–4. Three shrimp were sampled from each tank, and the mean value of the three shrimp within each tank was used for analysis. Each symbol represents one tank-level mean. Horizontal lines indicate the mean, and error bars indicate the standard deviation (SD) of three independent tanks (*n* = 3 independent tanks per treatment at each time point; *N* = 9 shrimp per treatment at each time point). Asterisks indicate significant differences between the PBS and VP groups at the same time point (* *p* < 0.05).

The mRNA transcript levels of immune-related genes were analyzed through quantitative real-time PCR. Firstly, we evaluated hemocyte subpopulations associated with pattern recognition receptor (PRR) genes (Figure 6). Expression of the pattern recognition receptor genes *lgbp* and *ctl-2* was compared between shrimps in the PBS and VP groups of the four hemocyte subpopulations (H1–H4) over 48 h. Expression of PRR genes in sorted hemocyte subpopulations of *L. vannamei* was differentially modulated following *V. parahaemolyticus* challenge. In the *lgbp* gene expression, H1, H3, and H4 showed no significant differences (*p* > 0.05) between VP and PBS at all sampling times. In contrast, *lgbp* expression in H2 was significantly higher (*p* < 0.05) in the VP group than PBS group at 12 h (Figure 6A–D). For *ctl-2*, H2 also showed significantly upregulated (*p* < 0.05) in the VP group compared to the PBS group at 6, 12 and 24 h, whereas no significant differences (*p* > 0.05) on the H1, H3, and H4 between both groups (Figure 6E–H). Overall, the result showed that VP infection primarily modulates PRR expression in the H2 subpopulation, with limited effects on the other hemocyte subpopulations.

**Figure 7:**
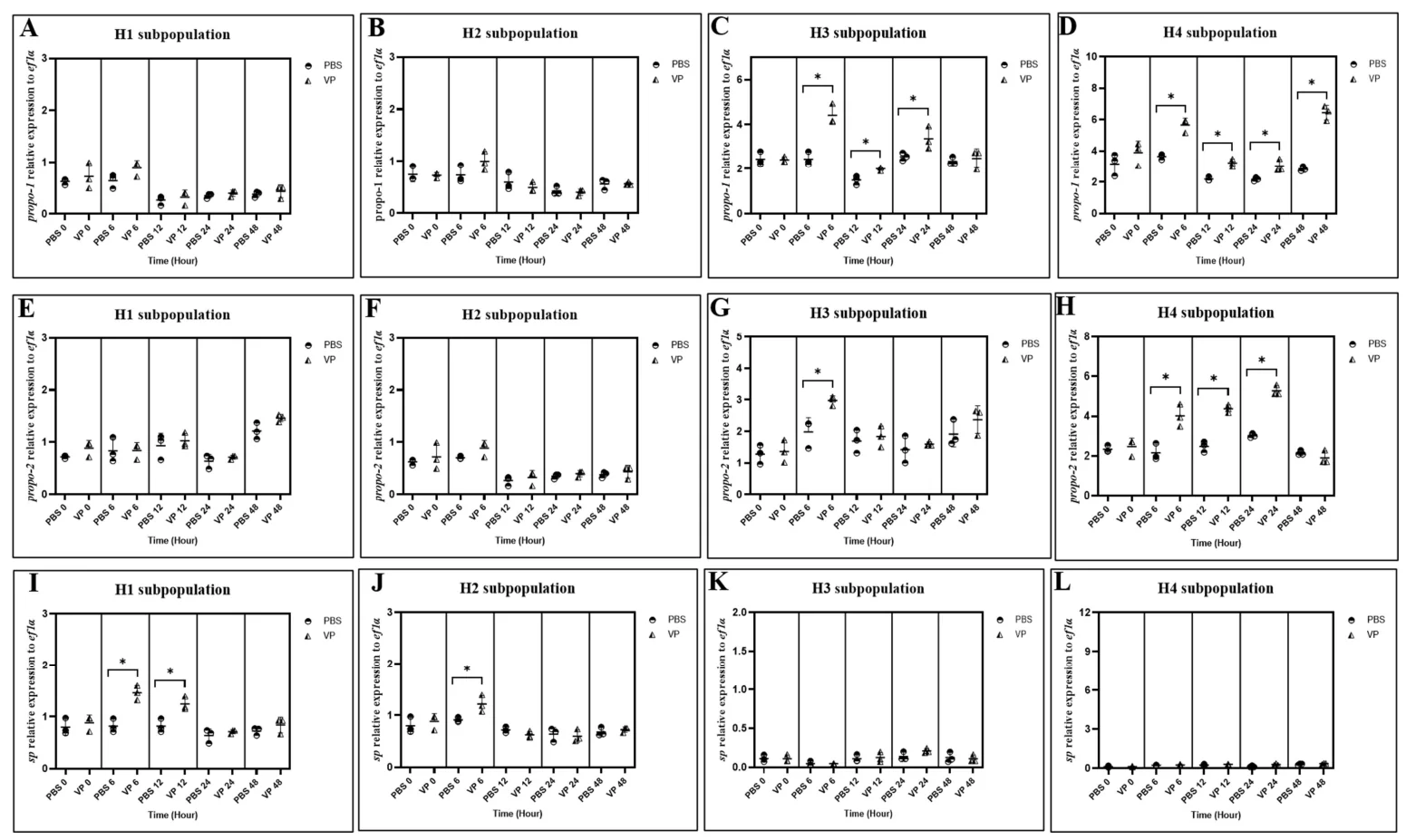
Expression of prophenoloxidase activation genes in hemocytes of *L. vannamei* following *V. parahaemolyticus* and PBS injection over 48 h. Expression levels of (**A**–**D**) Prophenoloxidase 1 (*propo-1*); (**E**–**H**) *propo-2*; and (**I**–**L**) Serine proteinase (*sp*) in H1, H2, H3, and H4, respectively. H1–4: hemocyte subpopulation 1–4. Three shrimp were sampled from each tank, and the mean value of the three shrimp within each tank was used for analysis. Each symbol represents one tank-level mean. Horizontal lines indicate the mean, and error bars indicate the standard deviation (SD) of three independent tanks (*n* = 3 independent tanks per treatment at each time point; *N* = 9 shrimp per treatment at each time point). Asterisks indicate significant differences between the PBS and VP groups at the same time point (* *p* < 0.05).

Expression of genes involved in prophenoloxidase activation (*propo-1*, *propo-2*, and *sp*) in hemocytes was differentially regulated after *V. parahaemolyticus* infection (Figure 7). In the *propo-1* gene expression, the H1 and H2 subpopulations showed no significant differences (*p* > 0.05) between the VP and PBS groups at all sampling times (Figure 7A,B). The H3 subpopulation in VP group showed a significant difference (*p* < 0.05) in *propo-1* expression levels compared with the PBS group at 6, 12, and 24 h, with a slight decrease at 12 h followed by an increase at 24 h (Figure 7C). The H4 subpopulation in VP group displayed a pattern similar to that of the H3 subpopulation, showing a significant difference (*p* < 0.05) in *propo-1* expression levels compared with the PBS group at 6, 12, 24, and 48 h, with slight decreases at 12 and 24 h followed by an increase at 48 h (Figure 7D). Meanwhile, for *propo-2* expression level showed there was no significant differences (*p* > 0.05) in H1 and H2 subpopulation in VP group compared to the PBS group (Figure 7E,F). H3 subpopulations exhibited significantly upregulated (*p* < 0.05) *propo-2* expression levels compared to the PBS group at 6 h (Figure 7G). In the H4 subpopulation, the VP group demonstrated significantly upregulated *propo-2* expression levels at 6,12, and 24 h (Figure 7H). For *sp* gene expression levels, the result showed that H1 had a significant increase (*p* < 0.05) compared to the PBS group at 6 and 12 h (Figure 7I). The H2 subpopulation in the VP group exhibited significantly upregulated *sp* gene expression levels at 6 h compared to the PBS group (Figure 7J). Meanwhile, the *sp* gene expression results showed that in the H3 and H4 groups there were no significant differences (*p* < 0.05) compared to the PBS group at all sampling times (Figure 7K,L). Overall, VP infection strongly induced *propo-1* and *propo-2* expression in the H3 and H4 subpopulations at nearly all sampling times, while *sp* gene expression was upregulated in the H1 and H2 subpopulations, particularly at 6 h after infection.

**Figure 8:**
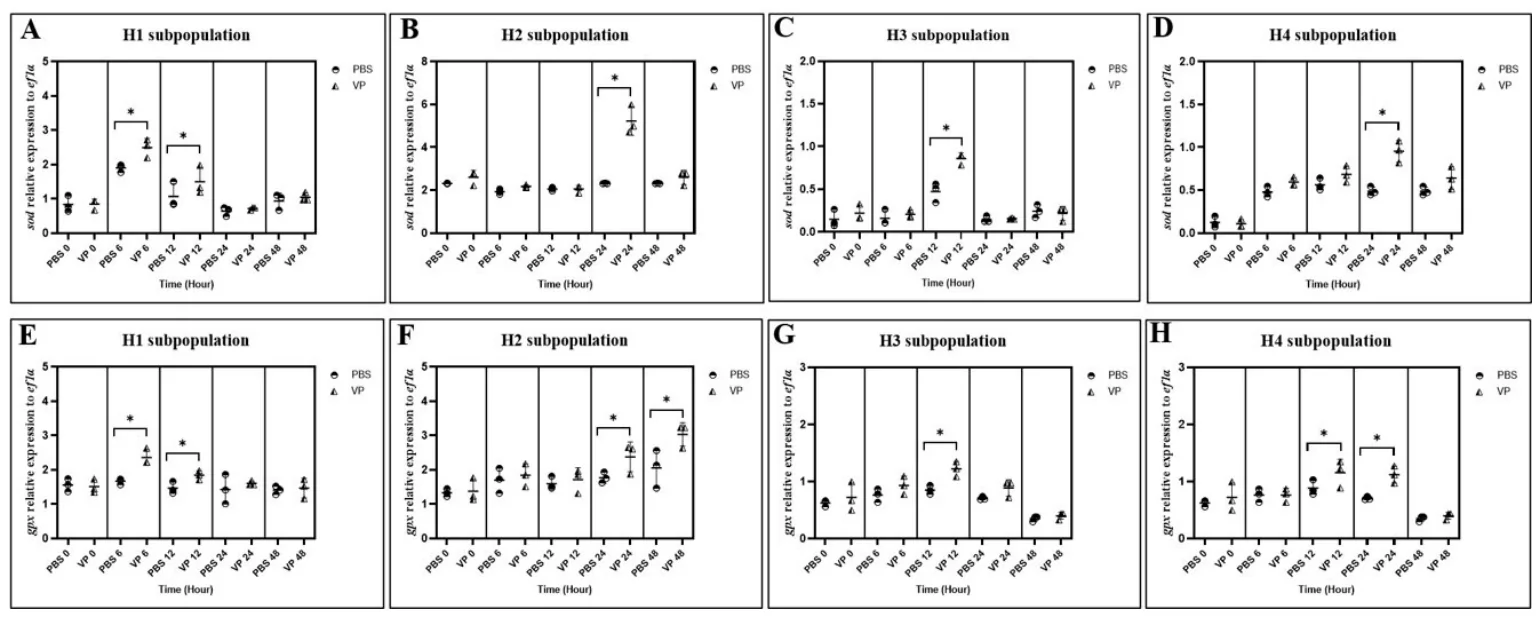
Expression of antioxidant defense genes in hemocytes of *L. vannamei* following *V. parahaemolyticus* and PBS injection over 48 h. Expression levels of (**A**–**D**) Superoxide dismutase (*sod*); and (**E**–**H**) Glutathione peroxidase (*gpx*) of H1, H2, H3, and H4, respectively. H1–4: hemocyte subpopulation 1–4. Three shrimp were sampled from each tank, and the mean value of the three shrimp within each tank was used for analysis. Each symbol represents one tank-level mean. Horizontal lines indicate the mean, and error bars indicate the standard deviation (SD) of three independent tanks (*n* = 3 independent tanks per treatment at each time point; *N* = 9 shrimp per treatment at each time point). Asterisks indicate significant differences between the PBS and VP groups at the same time point (* *p* < 0.05).

Expression of antioxidant defense genes such as *sod* and *gpx* was evaluated (Figure 8). In the H1 subpopulation, *sod* expression in the VP group was significantly higher than in the PBS group at 6 and 12 h (*p* < 0.05) but no significantly different at 24 and 48 h (*p* > 0.05), suggesting an early activation of antioxidant defenses during infection (Figure 8A). H2 subpopulation showed a higher expression level of *sod* at 24 h in the VP group compared to the PBS group (*p* < 0.05) (Figure 8B). H3 subpopulation demonstrated significantly upregulated (*p* < 0.05) expression level of *sod* at 12 h in the VP group compared to the PBS group (Figure 8C). H4 subpopulation exhibited significantly upregulated (*p* < 0.05) expression level of *sod* at 24 h in VP group compared to the PBS group (Figure 8D). For *gpx* expression level, the result showed that the H1 subpopulation had significantly upregulated (*p* < 0.05) in the VP group compared to the PBS group at 6 and 12 h (Figure 8E). The H2 subpopulation exhibited significant increased (*p* < 0.05) of *gpx* expression level in VP group compared to the PBS group at 24 and 48 h (Figure 8F). The H3 subpopulation showed significant upregulated (*p* < 0.05) of *gpx* expression level in VP group compared to the PBS group at 12 h (Figure 8G). The H4 subpopulation showed significantly increased (*p* < 0.05) of *gpx* expression levels in VP group compared to the PBS group at 12 and 24 h (Figure 8H). Overall, the *sod* and *gpx* were upregulated in all hemocyte subpopulations of the VP group, with each hemocyte subpopulation responding at different times but generally displaying coordinated increases in *sod* and *gpx* expression at the same sampling time.

**Figure 9:**
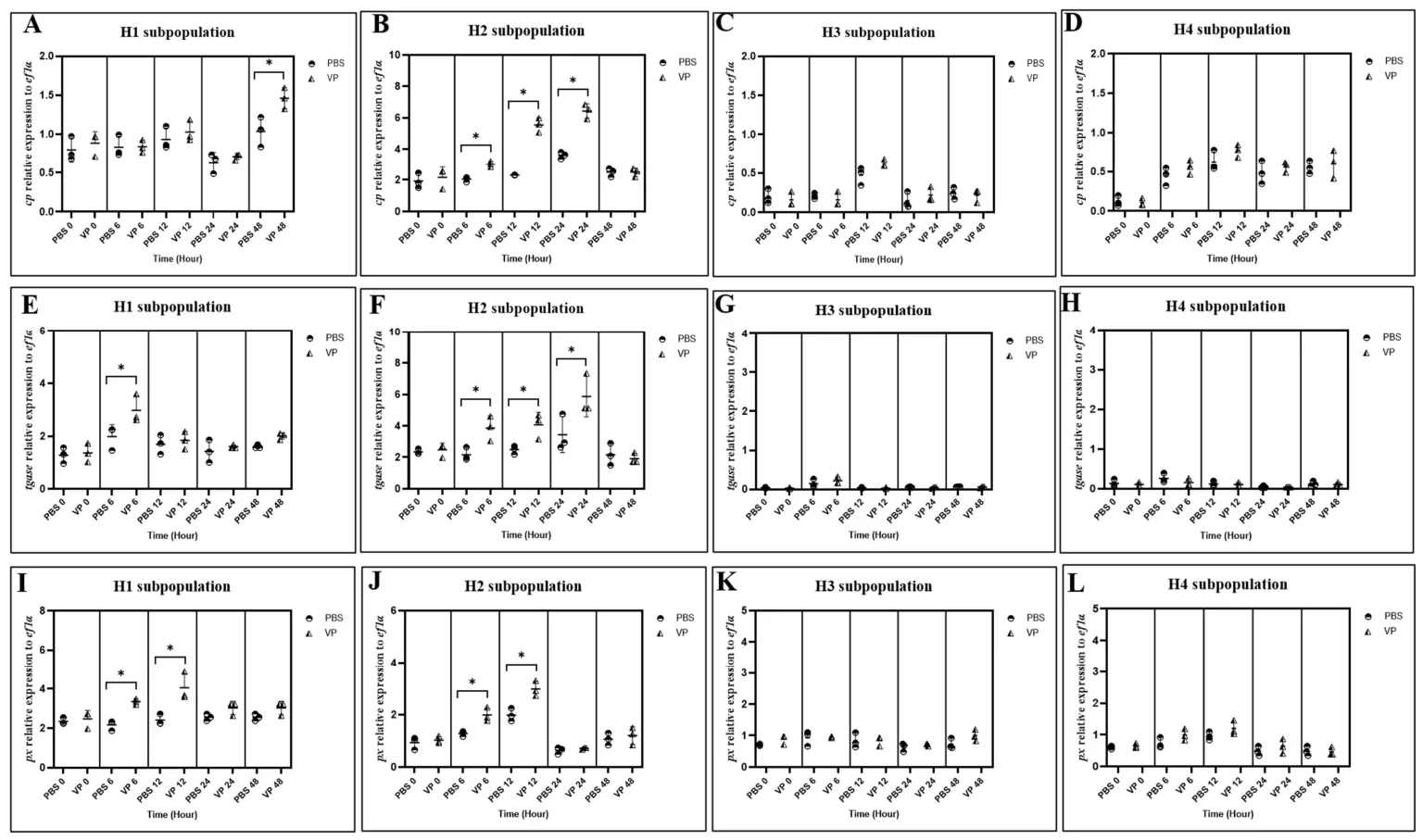
Expression of coagulation system genes in hemocytes of *L. vannamei* following *V. parahaemolyticus* and PBS injection over 48 h. Expression levels of (**A**–**D**) Clotting protein (*cp*); (**E**–**H**) Transglutaminase (*tgase*); and (**I**–**L**) Peroxinectin (*px*) of H1, H2, H3, and H4, respectively. H1–4: hemocyte subpopulation 1–4. Three shrimp were sampled from each tank, and the mean value of the three shrimp within each tank was used for analysis. Each symbol represents one tank-level mean. Horizontal lines indicate the mean, and error bars indicate the standard deviation (SD) of three independent tanks (*n* = 3 independent tanks per treatment at each time point; *N* = 9 shrimp per treatment at each time point). Asterisks indicate significant differences between the PBS and VP groups at the same time point (* *p* < 0.05).

Expression of coagulation-related genes such as *cp*, *tgase* and *px* was evaluated (Figure 9). In the *cp* gene, the H1 and H2 subpopulations in the VP group showed significantly higher expression than the PBS group at 48 h and 6, 12 and 24 h, respectively (*p* < 0.05) (Figure 9A,B). In contrast, *cp* expression in H3 and H4 did not differ significantly (*p* > 0.05) between VP and PBS at all sampling times (Figure 9C,D). For *tgase*, H1 and H2 showed significant upregulation in the VP group at 6 h and 6, 12 and 24 h, while H3 and H4 showed comparable levels between VP and PBS across all times examined (*p* < 0.05) (Figure 9E–H). The gene expression of *px* in the H1 and H2 subpopulations exhibited significant upregulation (*p* < 0.05) at 6–12 h in the VP group compared with PBS, whereas the H3 and H4 remained relatively stable and showed no marked differences between treatments (*p* > 0.05).

**Figure 10:**
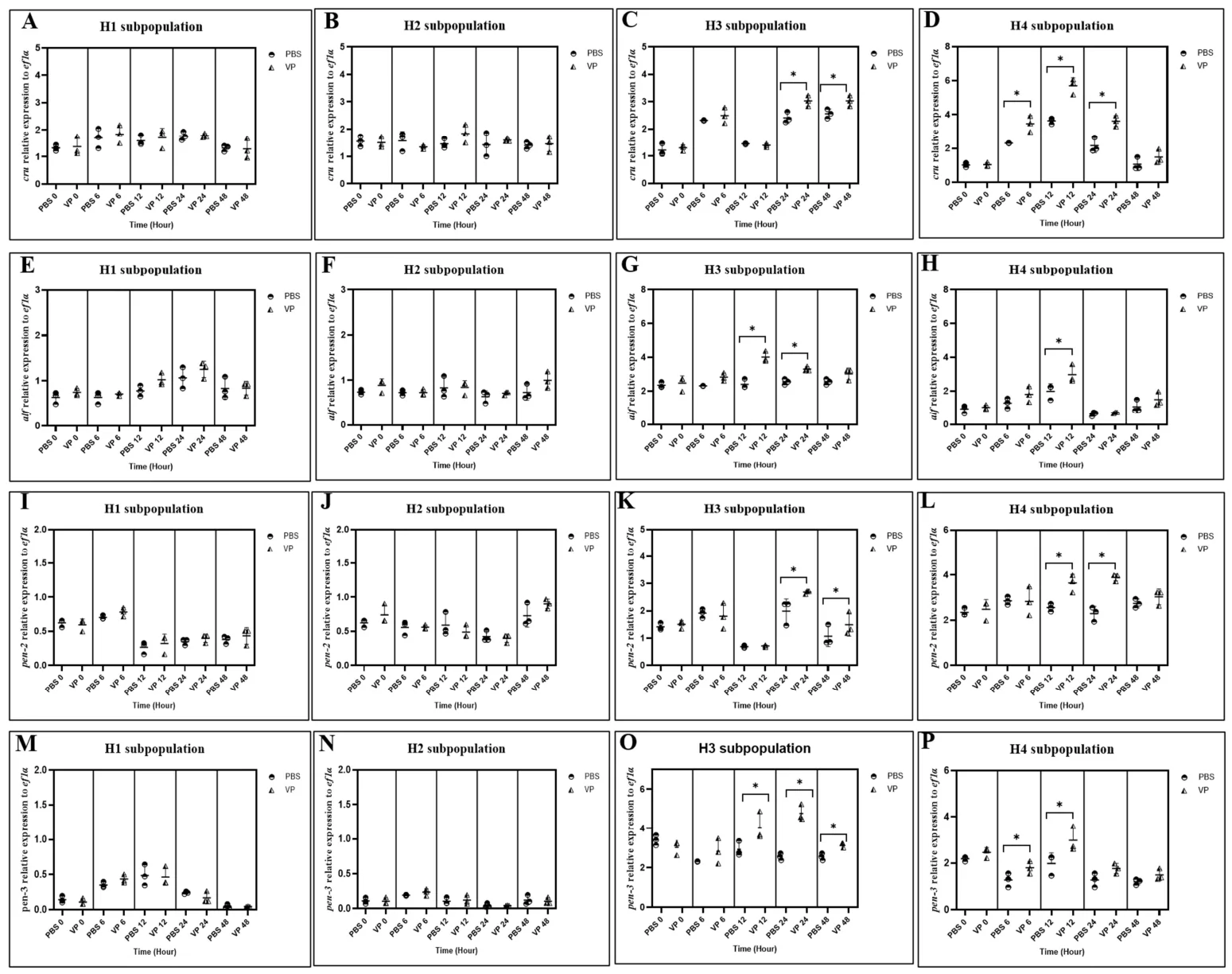
Expression of antimicrobial peptides (AMPs) genes in hemocytes of *L. vannamei* following *V. parahaemolyticus* and PBS injection over 48 h. Expression levels of (**A**–**D**) Crustin (*cru*); (**E**–**H**) Anti-lipopolysaccharide factor (*alf*); (**I**–**L**) Penaeidin 2 (*pen-2*); and (**M**–**P**) Penaeidin 3 (*pen-3*) of H1, H2, H3, and H4, respectively. H1–4: hemocyte subpopulation 1–4. Three shrimp were sampled from each tank, and the mean value of the three shrimp within each tank was used for analysis. Each symbol represents one tank-level mean. Horizontal lines indicate the mean, and error bars indicate the standard deviation (SD) of three independent tanks (*n* = 3 independent tanks per treatment at each time point; *N* = 9 shrimp per treatment at each time point). Asterisks indicate significant differences between the PBS and VP groups at the same time point (* *p* < 0.05).

Expression of antimicrobial peptide genes in *L. vannamei* hemocytes showed strong, subset-specific induction after *V. parahaemolyticus* challenge (Figure 10). In the crustin (*cru*) gene expression analysis, the H1 and H2 subpopulations showed no significant differences (*p* > 0.05) between the VP and PBS groups at all sampling time points. In contrast, the H3 and H4 subpopulations exhibited significantly higher expression levels (*p* < 0.05) in the VP group than in the PBS group at 24–48 h and 6–24 h, respectively (Figure 10A–D). For *alf*, H1 and H2 subpopulations showed no significant differences (*p* > 0.05) over 48 h, while H3 and H4 exhibited transient upregulation at intermediate time points (12–24 h and 12 h, respectively) in the VP group compared to the PBS group (*p* < 0.05) (Figure 10E–H). For *pen-2* and *pen-3* expression levels were predominantly upregulated (*p* < 0.05) in the H3 and H4 subpopulations compared with the PBS group, but with different time-dependent patterns: H3 was mainly induced during the late phase of infection (24–48 h), whereas H4 showed stronger upregulation in the early to middle phase of sampling time (12–24 h) (Figure 10I–P). Overall, these results suggest that AMPs are predominantly produced by H3 and H4 subpopulations during VP infection, highlighting these subpopulations as key effector cells in the antimicrobial response. Taken together, these results indicate that VP infection predominantly activates coagulation-related gene expression in the H1 and H2 hemocyte subpopulations, with no involvement of H3 and H4 in this response.

**Figure 11:**
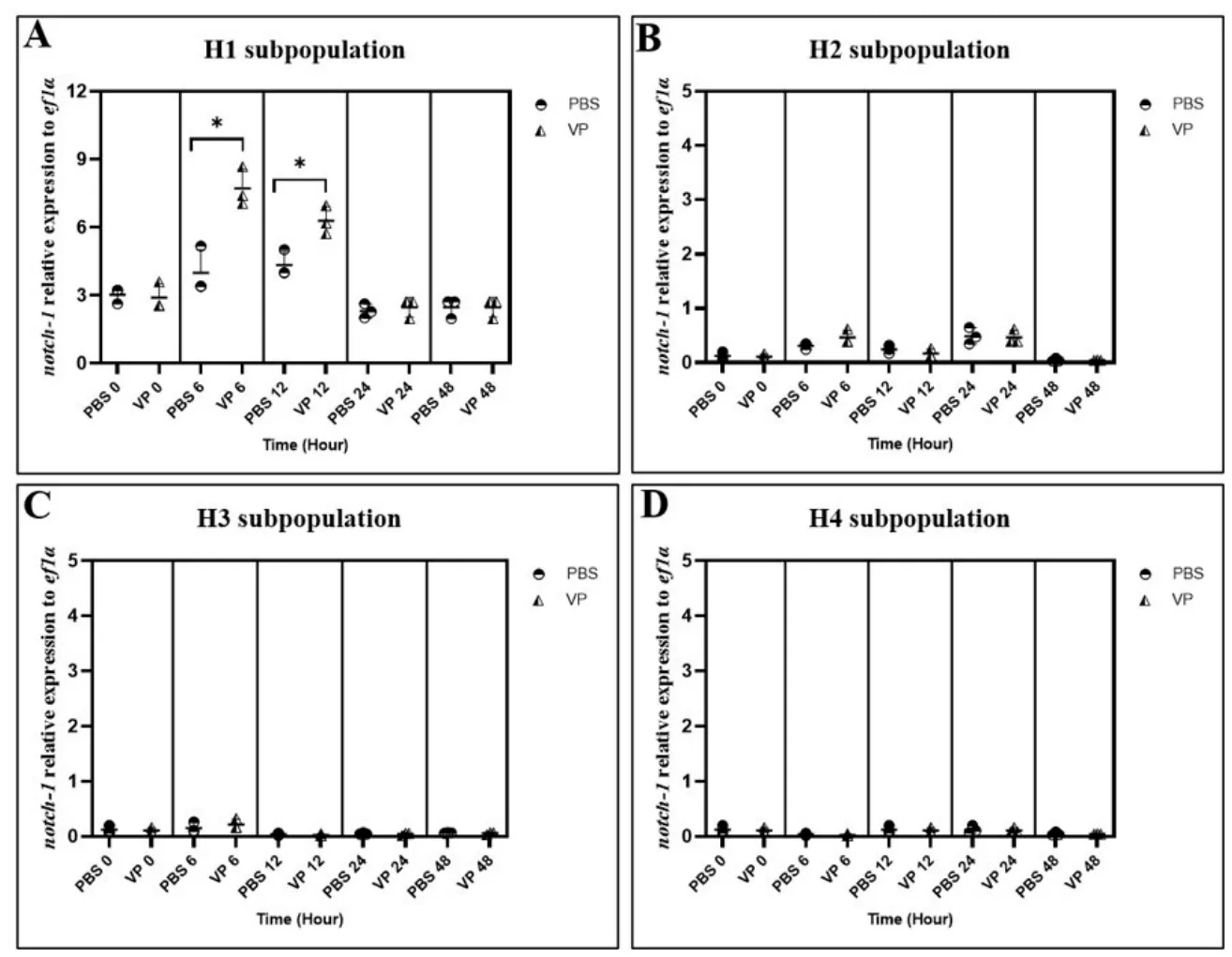
Expression of Neurogenic locus notch homolog protein-1 (*notch-1*) gene in hemocytes of *L. vannamei* following *V. parahaemolyticus* and PBS injeciton over 48 h. (**A**) Hemocyte subpopulation 1 (H1); (**B**) Hemocyte subpopulation 2 (H2); (**C**) Hemocyte subpopulation 3 (H3); (**D**) Hemocyte subpopulation 4 (H4). Three shrimp were sampled from each tank, and the mean value of the three shrimp within each tank was used for analysis. Each symbol represents one tank-level mean. Horizontal lines indicate the mean, and error bars indicate the standard deviation (SD) of three independent tanks (*n* = 3 independent tanks per treatment at each time point; *N* = 9 shrimp per treatment at each time point). Asterisks indicate significant differences between the PBS and VP groups at the same time point (* *p* < 0.05).

*notch-1* expression in *L. vannamei* hemocytes was mainly induced in the H1 subpopulation after *V. parahaemolyticus* challenge (Figure 11). In H1, VP group showed significantly (*p* < 0.05) higher *notch-1* transcript levels than PBS group at 6 and 12 h, followed by a decline toward baseline at late of sampling time (Figure 11A). Meanwhile, there were no significantly differences (*p* > 0.05) in the H2, H3 and H4 subpopulations compared to the PBS group in all sampling time (Figure 11B–D).

The overall response of the four hemocyte subpopulations to *V. parahaemolyticus* AHPND challenge is summarized in graphical abstract. Based on FSC-A/SSC-A characteristics, hemocytes were classified into four subpopulations: H1 (prohemocytes), H2 (hyalinocytes), H3 (semi-granulocytes), and H4 (granulocytes). Following VP challenge, the relative proportions of these hemocyte subpopulations showed time-dependent changes from 0 to 48 h post-infection. The functional analysis indicated distinct responses among the hemocyte subpopulations. At 6 h post-infection, *tgase* and *notch-1* showed higher expression in H1. At 12 h post-infection, PRR-related genes showed higher expression in H2, whereas AMP-related genes showed higher expression in H3 and H4. At 24 h post-infection, PRR and coagulation-related genes showed higher expression in H2. At 48 h post-infection, phenoloxidase-activating genes showed higher expression in H4. These findings indicate that the H1–H4 hemocyte subpopulations may have different functional roles during the immune response to VP challenge.

## 4. Discussion

Early mortality syndrome (EMS), also known as acute hepatopancreatic necrosis disease (AHPND), is a bacterial disease that has significantly impacted the shrimp aquaculture industry [28]. The present study provides an integrated analysis of non-specific immune responses and gene expression in FACS-sorted hemocyte subpopulations of *L. vannamei* following infection with a *V. parahaemolyticus* strain AHPND, thereby shedding light on the mechanisms underlying host responses during infection. Distinct hemocyte subpopulations were identified by FACS sorting and subsequently characterized by size and granularity as H1 (prohemocytes, PH), H2 (hyalinocytes, HC), H3 (semi-granulocytes, SGC), and H4 (granulocytes, GC) [5]. Our results show that VP-AHPND elicits a temporally coordinated, subtype-specific program in *L. vannamei* hemocytes, which is prohemocytes (H1) mount early proliferative and coagulation responses, followed by the activation of proPO and AMPs in semi-granulocytes and granulocytes (H3/H4). The result indicates that the temporal specialization of hemocyte functions points to precise cellular targets and timepoints for designing hemocyte-directed strategies to enhance disease resistance.

The time-course experiment of relative proportions of hemocyte subpopulations after *V. parahaemolyticus* challenge showed that each hemocyte contributes in a coordinated, time-dependent manner to the innate immune response in shrimp. The proportions of hemocyte subpopulations are influenced by seasonal variation, exposure to bacteria and viruses (such as WSSV and DIV1), and stimulation with microbial polysaccharides including LPS, laminarin, and β-1,3-glucan [8,29,30,31,32,33]. In this study, the results showed that VP infection altered hemocyte subpopulation percentages in a strongly time-dependent and subpopulation-specific manner in which time-dependent indicating that each subpopulation responded at different times rather than undergoing a uniform shift over 48 h. The increase in the H1 proportion at 6 h is consistent with previous single-cell RNA-seq findings, which reported that clusters 9 and 15 (prohemocytes) expand after WSSV infection. That study suggested that these PH are released from hematopoietic tissues to sustain overall hemocyte numbers and to supply precursors that can differentiate into diverse functional subsets in response to viral challenge [15]. The H1 subpopulation showed a transient increase at 6 h followed by a decline from 12–48 h, whereas H2 continued to rise throughout the experiment and H4 increased at 12–24 h, suggesting a shift from progenitor-like cells toward activated effector hemocytes over time. The previous study suggested that the increase in the immature hemocyte progenitor cluster (cluster 4) might result from an emergency hematopoiesis response mounted by the host to replenish hemocytes lost during YHV infection [34]. These dynamics are in line with single-cell RNA-seq data from DIV1-infected shrimp, where the proportions of clusters 0 and 5 (granulocyte-like, GC) and clusters 6 and 8 (hyalinocyte-like, HC) increased progressively during infection and were strongly associated with immune signaling, phagocytosis, the prophenoloxidase system, and antimicrobial peptide production [13]. There was also a decrease in the proportions of H1 after 12 h, H3 at 24 h, and H4 at 48 h in the VP group, which is consistent with previous findings that *V. parahaemolyticus* injection in shrimp causes a significant reduction in SGC, GC, and total hemocyte counts [12,35]. The proportional changes in the hemocyte subpopulation count may be due in part to altered gene expression in specific cells after exposure with the foreign substances [30,36], as discussed in more detail in the following sections.

In invertebrates, defense against infection is thought to rely exclusively on innate immunity. Medzhitov and Janeway (2000) explained that this system is activated when germline-encoded pattern-recognition receptors (PRRs), which are fundamentally different from vertebrate antibodies, detect invading pathogens [37]. Once engaged, these receptors rapidly initiate both humoral and cellular immune reactions. A previous study has identified several PRR families in invertebrates, such as Gram-negative binding proteins and lipopolysaccharide/β-1,3-glucan-binding proteins (LGBPs/GNBPs), as well as C-type lectins [38,39]. Lipopolysaccharide and β-1,3-glucan-binding protein (*lgbp*) is the initial defense in the immune system of white shrimp that can recognize the pathogens, then it could activate proPO [40,41]. The results showed that the PRR genes *lgbp* and *ctl-2* were predominantly upregulated in the H2 subpopulation, which is in accordance with a previous study showing that CTL mostly predominates in hyalinocytes [1,42]. This finding is consistent with a previous study reporting increased CTL expression in clusters 0 and 12, which were classified as early-stage differentiated cells (hyalinocytes) following WSSV infection, facilitating hemolymph coagulation to prevent WSSV diffusion in the hemolymph [15]. This result is consistent with the increase in H2 proportion during infection, mostly between 6–24 h.

When a certain pattern is recognized, the continue mechanism occurs that includes phagocytosis, melanization, and the creation of a hostile environment for the pathogen. The one indicator that was used for evaluating immune response in crustaceans were phagocytic activity and respiratory burst [43]. Invertebrates that encounter foreign pathogens, often release oxygen substances to protect from the infection respiratory burst is the rapid release of the reactive oxygen species (ROS), superoxide anion (O_2_^−^), and hydrogen peroxide (H_2_O) [44]. The present study data is consistent with our previous work under baseline conditions, in which H2 also exhibited the highest phagocytic activity among all hemocyte subpopulations [5]. In this study, phagocytic activity, O_2_^−^ production, *sod* expression level and *gpx* expression level after VP infection were concentrated primarily in the H2 (HC) at time point 6 and 24 h representing the early and late time of infection. This is in accordance with a previous study that showed that the HC from *Eriocheir sinensis* had involved in phagocytizing foreign particles after 6 hpi [45]. Our results also showed that after VP infection, phagocytosis occurred in H3 and H4, consistent with a previous study in *P. indicus* that demonstrated that SGC and GC are responsible for phagocytosing invading *V. alginolyticus* [46,47]. The higher H4 phagocytosis at 6 hpi may reflect an early cellular response to VP challenge, whereas the differences at 24 and 48 hpi indicate that enhanced phagocytosis was also evident at later sampling times. However, these observations do not establish a continuous enhancement throughout the sampling period. Further studies with more frequent sampling of H4 hemocytes are needed to clarify the timing and regulation of this response. Respiratory burst (O_2_^−^ production) is one of the main mechanisms by which phagocytized material can be eliminated [22]. In this study showed that the O_2_^−^ production was also increased in H3 and H4 subpopulations. This is consistent with previous findings that, after LPS infection, ROS levels in *P. monodon* are significantly increased in SGC and GC from several hours post-injection and persist into the later phase [9]. *sod* helps limit excess superoxide anion during phagocytosis, while *gpx* and catalase break down the resulting hydrogen peroxide, together forming an antioxidant self-protection system [48,49]. The patterns of *sod* and *gpx* expression indicate that *gpx* expression depends on the *sod* expression. These findings suggest that antioxidant defenses after VP infection are activated in the hemocyte subpopulation-specific manner.

Phenoloxidase (PO) activity is a crucial enzyme in melanin synthesis, where the proPO is activated by phenoloxidase (PO) when reacting with zymosan, lipopolysaccharide (LPS), calcium ions, and trypsin [50]. ProPO is activated to produce PO and is released from the hemocyte to the plasma, then synthesizes melanin, in a process in which phenol interacts with quinone, which is then polymerized to form melanin [51]. Classical work in penaeid shrimp has generally attributed the main proPO and melanization functions to SGC and GC, while hyalinocytes are considered primarily phagocytic with limited PO involvement [50,52,53]. Bulk-hemocyte studies under *Vibrio* challenge have reported increased PO activity and upregulation of proPO system genes [35,46], but did not show these changes to specific subpopulations. The present study showed that, after *V. parahaemolyticus* infection, *propo-1* and *propo-2* are mainly upregulated in H3 and H4 (with the highest *propo-1* upregulation in H4 at 48 h), indicating that later steps of the PO cascade are concentrated in the semi-granular and granular hemocytes H3 and H4. This is consistent with previous studies showing that, during IMNV infection in *L. vannamei* and after LPS injection in *P. monodon*, foreign particles trigger SGC and GC activation and release components of the proPO system [9,54,55]. The marked increase in *propo-1* in H4 at 48 h coincided with the decrease in the H4 proportion, suggesting that H4 cells underwent degranulation to release components of the proPO system [55]. In contrast, our results showed that *sp* expression increased mainly in H1 and H2 (PH and HC) at early sampling time. Although serine proteinases in *L. vannamei* hemocytes have previously been reported to be mainly expressed in GC [29,42,56], we found that *sp* is strongly expressed in H1 subpopulation (PH) but exhibited relatively low expression in H4 subpopulation; however, given the limited sampling time, we cannot exclude the possibility that higher expression occurred at other times between or over 48 h. Serine proteinases are a key activator of the proteolytic cascade, which is essential for regulating the proPO system and establishing the melanization pathway in vertebrates [57]. These findings showed time-dependent differences in *sp* and *propo* gene-expression patterns among the FACS-defined hemocyte subpopulations following VP-AHPND exposure. *sp* transcript levels changed primarily in H1 at early sampling time points, whereas *propo* transcript levels changed predominantly in H3 and H4 at later time points.

Crustacean blood clot is formed by the polymerization of a plasma protein catalyzed by a Ca^2+^ dependent transglutaminase (TGase), which is released from hemocytes under foreign particle stimulus or tissue damage [58]. In *L. vannamei,* TGase is mostly found in hyalinocytes, although some gene expression has also been observed in semi-granulocytes and granulocytes [59]. The clotting system in *L. vannamei* belongs to the type C category, in which the hemocytes are rapidly lysed, and the plasma is clotted immediately, resulting in low cell aggregation [60,61]. Peroxinectin (*px*) is a known cell adhesion factor that is synthesized and stored in secretory granules of semi-granular and granular cells in an inactive form, released in response to stimulus, and activated outside the cells to mediate hemocyte attachment and spreading [62]. *tgase* and *cp* reported to be involved in the blood coagulation system of shrimp [63]. The present data indicate that H1 and H2 are the key coagulation-competent subpopulations during VP infection, but they act at different stages and through partially distinct mechanisms: H1 responds very early via upregulation of *px* and *tgase*, while H2 dominates later via robust induction of *cp* and *tgase*. This finding aligns with previous studies in *P. vannamei*, where HCs were identified as the primary hemocyte type responsible for blood clotting and exhibited high *tgase* expression, as determined by single-cell RNA sequencing [1,2].

Antimicrobial peptides (AMPs) are important elements against pathogens. Shrimp AMPs consist of penaeidins, crustins, lysozyme, and anti-lipopolysaccharide factor that are produced and stored in hemocytes [64,65,66]. Responses of diverse AMPs against microbial infection in shrimp have been reported. Penaeidins and crustins are two main antimicrobial peptides in penaeid shrimps [67]. Crustin consists of a whey acid protein (WAP) domain, which inhibits microbial growth [68,69]. The present study found that AMP profiles support a model in which H3 and H4 constitute the core antimicrobial effector hemocytes, with H4 acting as the major AMPs (*cru*, *alf*, *pen-2*) and H3 providing substantial support (*cru*, *pen-2*) and a more prominent role for *pen-3* at most of the time sampling. Our results follow a similar pattern to those in *E. sinensis* infected with *Spiroplasma eriocheiris*, where increased expression of AMPs (alf and lysozyme) was observed specifically in the Hem1 and Hem5 clusters (the cluster in which AMPs are most abundantly identified), indicating that these cell types participate in host elimination of invading *S. eriocheiris* [70]. Moreover, the previous study showed that the GC had upregulated crustin expression in Hyas araneus after 24 h exposure to *Listonella anguillarum* [71].

The Notch signaling pathway, first characterized in *Drosophila*, is evolutionarily conserved from invertebrates to vertebrates and has been implicated in regulating cell proliferation, differentiation, survival, and apoptosis across diverse biological systems [72,73,74,75]. In *Drosophila*, Notch signaling has been implicated in crystal-cell differentiation and coagulation-related functions [76]. In the present study, the H1 subpopulation showed relatively higher *notch-1* transcript levels following VP-AHPND exposure. Previous studies have proposed that *notch-1* may be associated with hemocyte-cell states or differentiation-related processes in *L. vannamei* [77]. In the present study, the relative proportion of the FACS-defined H1 subpopulation changed over time following VP-AHPND exposure, and *notch-1* transcript levels were detected predominantly in H1 at early sampling time points. Previous studies have reported *Notch*-associated expression patterns in specific *L. vannamei* hemocyte clusters following WSSV infection and showed the reduction in their proportions of hemocyte [15,78]. Together, these observations suggest that the relative distribution of H1 hemocyte subpopulation and *notch-1* expression may be associated with early hemocyte responses to VP-AHPND exposure. However, because H1 was operationally defined using FSC-A/SSC-A characteristics and because total/absolute hemocyte counts, cell lineage, proliferation, differentiation, migration, and Notch-pathway activity were not measured, these results do not establish that H1 represents a progenitor population or that *notch-1* drives hemocyte differentiation or mobilization. Further functional validation is needed.

The FACS-defined H1 subpopulation showed relatively high *notch-1* transcript levels and time-dependent changes in the expression of *tgase* and *px* following VP-AHPND exposure. Changes in phenoloxidase activity were also observed during the experimental period. H2 showed time-dependent changes in the transcript levels of *lgbp*, *ctl-2*, *cp*, and *tgase*. H3 and H4 displayed changes in the expression of propo-related genes and antimicrobial peptide genes, including *cru*, *alf*, *pen-2*, and *pen-3*, while *sod* and *gpx* expression patterns were also assessed across the FACS-defined hemocyte subpopulations. Together, these findings describe temporal associations between VP-AHPND exposure, relative distributions of FACS-defined hemocyte subpopulations, non-specific immune parameters, and the expression of selected immune-related genes. However, the present study did not directly examine upstream signaling pathways, which warrants further investigation. In addition, the observed decreases in the relative proportions of selected FACS-defined hemocyte populations cannot be attributed to apoptosis based on the present data. Although apoptosis in shrimp hemocytes has been reported following exposure to *pirB*, apoptosis-related markers were not measured, and direct apoptosis assays, including caspase-expression analysis, Annexin V staining, and TUNEL labeling, were not performed in this study. Consequently, the observed proportional changes may reflect several unmeasured processes, including possible apoptosis, degranulation, tissue migration, cellular consumption, or changes in the relative proportions of other hemocyte populations. Accordingly, the observed gene-expression patterns should be interpreted as temporal associations with experimental VP-AHPND exposure rather than direct evidence that individual H1–H4 populations perform specific immune functions or act sequentially during the immune response. Further studies evaluating key signaling components, including the TLR/NF-κB pathway, together with functional validation approaches such as gene knockdown or pathway-inhibition experiments, are needed to clarify the mechanistic relationships among hemocyte subpopulations, pathogen recognition, and downstream immune responses. This study provides descriptive evidence of hemocyte-subpopulation-associated responses following experimental VP-AHPND exposure. Future studies should integrate absolute hemocyte counts, direct apoptosis and functional cell assays, cell-lineage validation, protein-level analyses, and gene-specific perturbation experiments.

A limitation of this study is that mortality was not systematically quantified under the present experimental conditions. Although some mortality was observed after VP-AHPND challenge, the challenge experiments were not performed synchronously, and standardized mortality records were not available. Therefore, a reliable mortality rate or Kaplan-Meier survival analysis could not be reconstructed. Consequently, the relationship between the challenge dose and mortality patterns could not be established. The four hemocyte subpopulations may have developmental relationships rather than representing entirely independent cell types. In *M. rosenbergii*, pseudotime analysis suggested that DIV1 infection accelerated differentiation of prohemocytes and semi-granulocytes toward granulocytes and hyalinocytes [13]. Evidence from crayfish also indicates that immune stimulation can influence progenitor-cell differentiation through hematopoietic factors such as astakine [79]. These findings support the possibility that pathogen challenge may modulate hemocyte maturation. However, these findings do not establish whether one hemocyte subpopulation can differentiate into another in response to pathogen challenge. Cell-tracking studies combined with molecular characterization are needed to clarify the developmental relationships and differentiation trajectories of these hemocyte populations.

## 5. Conclusions

In conclusion, experimental VP-AHPND exposure was associated with time-dependent changes in the relative proportions of FACS-defined hemocyte populations, non-specific immune parameters, and immune-gene expression. H1 (prohemocyte) cells showed increased transcript levels of *tgase*, *px*, and *sp* at early sampling time points, together with a transient increase in measured phenoloxidase (PO) activity. H2 (hyalinocyte) cells showed time-dependent changes in the transcript levels of *lgbp*, *ctl-2*, *cp*, and *tgase*. H3 (semi-granulocyte) and H4 (granulocyte) cells displayed comparatively higher measured PO activity and increased transcript levels of *propo-1*, *propo-2*, and several antimicrobial-peptide genes, including *cru*, *alf*, *pen-2*, and *pen-3*, particularly between 12 and 48 h post-injection. Because H1–H4 were operationally defined by FSC-A/SSC-A flow-cytometric gating and their relative proportions were measured after cell-density normalization, these findings describe temporal associations rather than absolute changes in hemocyte abundance, validated cell-lineage identities, or causal immune mechanisms. Further functional experiments are required to determine the specific roles of these hemocyte populations during naturally acquired AHPND.

## Figures and Tables

**Table 1:** Primers used for immune-related gene expressions.

	Gene	Forward (5′→3′)	Reverse (5′→3′)	GenBank ID
Internal factor (Housekeeping gene)	Elongation factor 1-α (*ef1α*)	ATGGTTGTCAACTTTGCCCC	TTGACCTCCTTGATCACACC	GU136229
Pattern recognition receptors (PRRs)	C-Type Lectin-2 (*ctl-2*)	CCACTACTTCTACATTAGCGACGAGG	TCGAAAAGCATCAGGTGCTCAC	OQ470535.1
	LPS-β-1,3-glucan binding protein (*lgbp*)	CGGCAACCAGTACGGAGGAAC	GTGGAAATCATCGGCGAAGGAG	AY723297
ProPO activating	Prophenoloxidase-1 (*propo-1*)	ACGTCACTTCCGGCAAGCGA	CCTCCTTGTGAGCGTTGTCAGG	AY723296
	*propo-2*	ACCACTGGCACTGGCACCTCGTCTA	TCGCCAGTTCTCGAGCTTCTGCAC	EU373096
	Serine proteinase (*sp*)	CGTCGTTAGGTTAAGTGCGTTCT	TTTCAGCGCATTAAGACGTGTT	AY368151
Antioxidant defenses	Superoxidase dismutase (*sod*)	ATCCACCACACAAAGCATCA	AGCTCTCGTCAATGGCTTGT	DQ005531
	Glutathione peroxidase (*gpx*)	TTTTTCCGTGCAAAAAGGAC	TAATACGCGATGCCCCTAAC	AY973252
Clotting protein	Clotting protein (*cp*)	TCTTTGCGCAGTTGGTGATC	TGAGGTGACCGAGTGCAAAA	DQ984182
	Transglutaminase (*tgase*)	CGAGATGTACGAGCTGGTCGAG	GGACGAGGACCGAAGGAGAAG	EF081004.1
	Peroxinectin (*px*)	ATCCAGCAGCCAGGTATG	CAGACTCATCAGATCCATTCC	KC708021
Antimicrobial peptides	Crustin (*cru*)	GAGGGTCAAGCCTACTGCTG	ACTTATCGAGGCCAGCACAC	AY488497
	Anti-LPS factor (*alf*)	CTGTGGAGGAACGAGGAGAC	CCACCGCTTAGCATCTTGTT	GE325385
	Penaeidin2 (*pen-2*)	TCGTGGTCTGCCTGGTCTT	CAGGTCTGAACGGTGGTCTTC	Y14925
	Penaeidin3 (*pen-3*)	CACCCTTCGTGAGACCTTTG	AATATCCCTTTCCCACGTGAC	AF390139
Cell proliferation	Neurogenic locus notch homolog protein 1 (*notch-1*)	GTTGCCCAAGATGCTCCTCC	CCTTGGCCTTGTGCGTTG	XM_070142666.1

## Data Availability

The datasets analyzed in this study are available upon reasonable request.

## Supplementary Materials

The following supporting information can be downloaded at: https://www.mdpi.com/article/10.3390/ani16203209/s1, Figure S1: Pathogen screening of shrimp samples.
