# Supplementary material for: Comprehensive Non-Specific Immune and Gene Expression Profiling of FACS-Sorted Hemocyte Subpopulations in Litopenaeus vannamei After Vibrio parahaemolyticus (AHPND Strain) Infection
Source: Animals (Basel). 2026 Oct 12;16(20):3209. doi: 10.3390/ani16203209 (PMC13700786; doi:10.3390/ani16203209)
Supplement: Supplementary file 1 [file animals-16-03209-s001.zip › animals-4597285-supplementary.pdf]

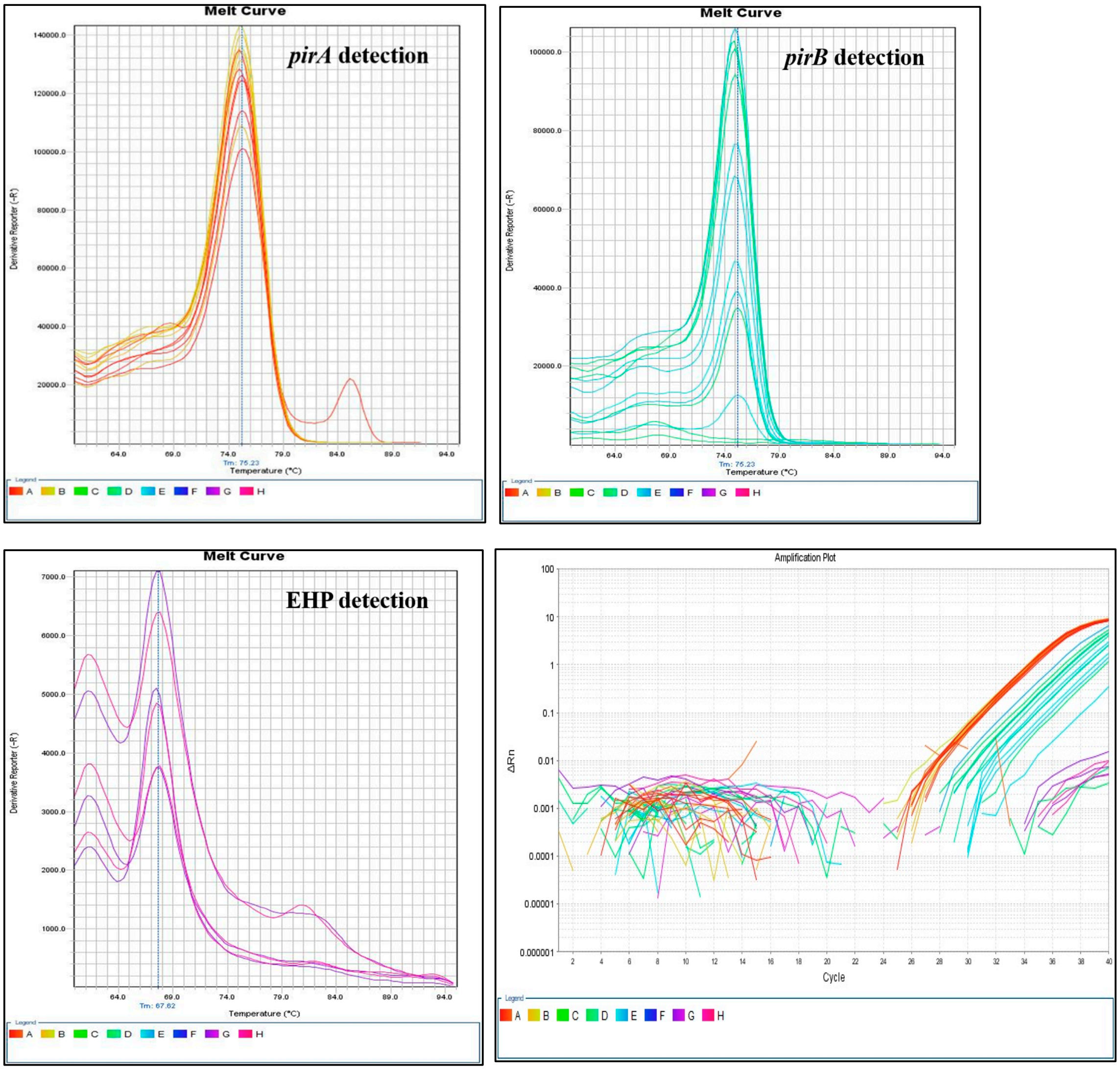

Supplementary Figure S1: Pathogen screening of shrimp samples. Representative results of screening for *Vibrio parahaemolyticus* toxin genes (*pirA* and *pirB*) and *Enterocytozoon hepatopenaei* (EHP). Melting-curve analyses of the *pirA*, *pirB*, and EHP targets, together with the corresponding qPCR amplification plot, are shown. Hemocyte and hepatopancreas samples from five randomly selected shrimp were screened for the AHPND-associated *pirA* and *pirB* genes and for EHP. *pirA*, *pirB*, and EHP refer to the target assays used for pathogen screening.
